# Linking Surface Wettability to Interfacial Thermal Transport at Ti–Water Interfaces: A Molecular Dynamics Study

**DOI:** 10.3390/mi17080972

**Published:** 2026-08-18

**Authors:** Haoming Huang, Xi Wang, Ming Ma, Shan Qing, Zhumei Luo, Xiaoyan Huang, Jing Zhang, Xiaohui Zhang

**Affiliations:** 1 State Key Laboratory of Complex Nonferrous Metal Resources Clean Utilization, School of Metallurgical and Energy Engineering, Kunming University of Science and Technology, Kunming 650093, China; 2 School of Metallurgical and Energy Engineering, Kunming University of Science and Technology, Kunming 650093, China; 3 Yunnan Electric Power Experimental Research Institute (Group) Co., Ltd., Kunming 650217, China; 4 School of Nationality Educators, Qinghai Normal University, Xining 810016, China

**Keywords:** wettability, molecular dynamics, interfacial thermal conductance, solid–liquid interface, vibrational density of states

## Abstract

Solid–liquid interfacial heat transfer plays a key role in microelectronic devices, energy systems, and liquid cooling technologies. However, the vibrational mismatch at solid–liquid interfaces produces an interfacial thermal resistance (ITR) that limits the heat-dissipation efficiency. Herein, molecular dynamics (MD) simulations were used to study the regulation of heat transfer at Ti–water interfaces by the Ti-O interaction strength. As the interaction strength increased, the Ti surface changed from strongly hydrophobic to complete wetting, with the contact angle spanning 153° to θ < 5° over the full droplet series. Over the range where the interfacial thermal conductance (ITC) was computed, the contact angle decreased from about 143° to 15°, and the ITC increased from 35.71 ± 4.26 to 231.97 ± 14.17 MW/m^2^·K. This increase originated from changes in the interfacial water structure, as the stronger interaction led to a denser and more ordered near-wall water structure that became more tightly bound to the surface, which enhanced the solid–liquid vibrational coupling. From the phonon perspective, the spectral overlap increased by only about 2%, from 0.01973 to 0.02008 THz^−1^, while the ITC increased by a factor of 6.5, indicating that the spectral overlap is not the controlling factor. Instead, the phonon lifetime of the interfacial Ti shortened markedly while the phonon heat capacity remained stable, showing that the enhancement originates from the stronger interfacial coupling rather than from an increase in the spectral overlap. This work clarifies how wettability regulates the microscopic structure of interfacial water and interfacial vibrational coupling, and provides a basis for understanding heat transfer at metal–water interfaces.

## 1. Introduction

Microelectronic devices, energy systems, and optoelectronics are moving toward higher power densities and greater integration, while device sizes keep shrinking. This raises the internal heat flux, and heat dissipation has become a key bottleneck for device performance and reliability [1,2]. As device dimensions decrease to the micro- and nanoscale, interfaces account for a growing fraction of the overall heat-transfer path. Consequently, interfacial thermal resistance (ITR) may become dominant over bulk thermal resistance and limit heat-dissipation efficiency [3]. Solid–liquid interfaces play a key role in liquid cooling, two-phase heat transfer, and immersion cooling [4]. However, the highly ordered lattice vibrations in solids and the disordered molecular thermal motion in liquids do not couple efficiently across the interface. This mismatch impedes energy exchange and gives rise to ITR, which becomes particularly limiting at nanoscale solid–liquid interfaces [5,6]. In reality, ITR is jointly governed by multiple factors such as interfacial structure, wettability, and bonding strength, but the coupling mechanism among these factors remains unclear. Clarifying this mechanism is a key prerequisite for designing interfaces with low thermal resistance [7,8].

Because energy transfer across solid–liquid interfaces occurs at the nanoscale, the underlying microscopic processes are difficult to observe directly in experiments. Molecular dynamics (MD) simulations have therefore become an important tool for investigating solid–liquid interfacial heat transfer. In recent years, MD simulations have been applied to various solid–liquid interfaces, including surfaces with different wettabilities [9], surfaces modified with self-assembled monolayers [10], and water interfaces with metals [11], graphite [12], graphene [13], SiC [14], alumina [15], and SiO_2_ [16]. Among these factors, surface wettability and surface chemistry have drawn the most attention, mainly because of their influence on interfacial thermal conductance (ITC). Gonçalves et al. [17] found that increasing the hydrophobicity of silica surfaces disrupted the interfacial hydrogen-bond network and significantly increased ITR. These studies indicate that stronger coupling between solids and liquids generally improves interfacial heat transfer. However, wettability alone may not be a sufficient predictor of ITC. Ramos-Alvarado et al. [18] compared bare silicon surfaces with graphene-coated silicon surfaces and found that interfaces with similar wettabilities could still exhibit substantially different ITC. Their results indicated that the density depletion length of the interfacial liquid provided a more accurate characterization of this difference. Mandrolko et al. [19] studied SiO_2_–water interfaces modified with different functional groups, further pointing to the role of interfacial liquid structures. They found that surface functionalization changed both the interfacial adhesion and the orientation and density depletion length of interfacial water, which in turn affected interfacial heat transfer. In addition to wettability, surface chemistry, nanoscale roughness, heat-flow direction, crystallographic orientation, and surface termination can also affect ITC [20].

These influences on ITC can be traced largely to the distinct microscopic state of the liquid near a solid surface. Compared with bulk liquids, interfacial liquids are constrained by surface potential fields. Their density distributions, hydrogen-bond networks, molecular mobility, and spatial organization may therefore change [21,22,23]. Motokawa et al. [24] proposed the radial density depletion length for Si–water interfaces containing atomic-scale surface structures. They showed that the local extent of liquid depletion could reflect interfacial heat-transfer capability. Ma et al. [25] studied Fe nanoparticle–water interfaces and observed a double-peaked interfacial water-density profile. They reported that the peak density increased with wettability, indicating enhanced accumulation of interfacial water molecules. In a related heterointerface system, Wang et al. [26] found that adsorbed water molecules formed dense interfacial channels at interfaces between gold and a metal–organic framework. These channels activated high-frequency lattice vibrations and provided additional pathways for heat transfer. The microscopic structure of the interfacial liquid thus stands out as a key factor controlling interfacial heat transfer.

Classical models have often related ITC to wettability, for example through the scaling between ITC and the work of adhesion, or the relation between ITC and (1 + cos θ) established by Shenogina et al. [27]. At a more microscopic level, this heat transfer depends on the vibrational coupling between interfacial atoms and molecules, which is commonly assessed through the overlap of their vibrational density of states (VDOS). This overlap alone, however, does not capture the full energy-exchange process. El-Rifai et al. [28] found that conventional VDOS overlap was insufficient to explain the transition between different ITC regimes as the solid–liquid interaction strength increased. Through spectral heat-current analysis, they further showed that the emergence of solid-like energy transport in the interfacial liquid altered the interfacial heat-transfer behavior. Lin et al. [29] showed that phonon–phonon coupling and interfacial binding jointly influenced interfacial thermal transport. In addition to frequency matching, the contributions of vibrational modes in different directions should also be considered. El-Rifai et al. [30] investigated solid–liquid interfacial heat transfer in the presence of a meniscus using spectral heat-current decomposition. They found that, under low-to-moderate wetting conditions, the enhancement in ITC mainly resulted from stronger coupling of vibrations normal to the interface. As wettability increased further, the utilization of in-plane vibrational modes also rose significantly. Recent studies have also shown that interfacial composition and molecular structure can alter vibrational energy exchange. Ma et al. [31] studied gold–water interfaces modified with self-assembled monolayers and identified interfacial interaction energy and vibrational spectral coupling strength as effective metrics for screening interfaces with high ITC. Zhang et al. [32] investigated heat transfer between liquid gallium and diamond or copper surfaces. They found that increasing temperature enhanced interfacial vibrational coupling and reduced ITR, while the difference in heat transfer between the two interfaces was mainly associated with the degree of vibrational mode matching. Solid–liquid interfacial heat transfer thus depends not on any single factor but on the interplay among interfacial structure, dynamics, and vibrational energy exchange. For metal–water interfaces, the connection between the wetting state, the microscopic structure of interfacial water, and the resulting vibrational coupling remains to be fully clarified.

In this study, we use molecular dynamics simulations to investigate heat transfer across Ti–water interfaces with different wetting states. By varying the Ti-O interaction strength, we construct a series of solid–liquid interfaces ranging from hydrophobic to hydrophilic conditions and characterize their wettability and interfacial binding strength using the contact angle and work of adhesion. On this basis, we calculate ITC using nonequilibrium molecular dynamics (NEMD) simulations and analyze short-range solid–liquid correlations and interfacial water enrichment through the Ti-O radial distribution function (RDF) and water-density distribution. We further examine the evolution of interfacial water structure using molecular orientation distributions, the four-body structural order parameter (*F*_4_), and the density depletion length. Finally, the phonon-mediated energy transfer across the solid–liquid interface is examined. The degree of vibrational coupling is quantified by the VDOS overlap, while the envelope decay time, phonon lifetime, and phonon heat capacity are used to assess the efficiency of interfacial energy transfer. This work clarifies the effect of wettability on the microscopic state of interfacial water and on vibrational energy exchange, and shows that the total spectral overlap is not the dominant factor controlling the wettability-dependent interfacial heat transfer, providing a basis for understanding heat transfer at metal–water interfaces.

## 2. Simulation Methods

### 2.1. Ti–Water Interface Construction

**Figure 1:**
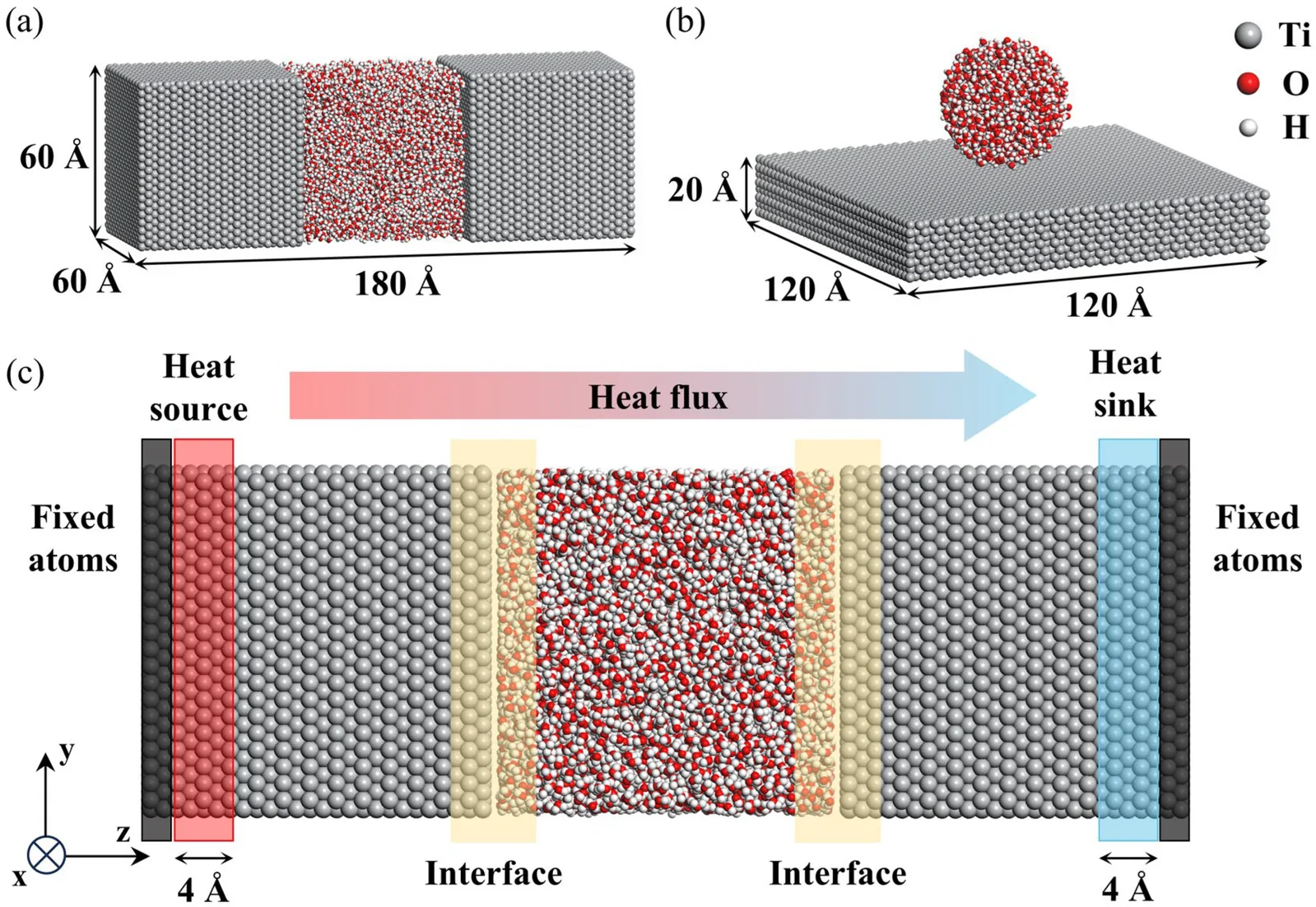
Simulation models. (**a**) Physical model of interfacial heat transfer. (**b**) Physical model of water wetting on the Ti surface. (**c**) Schematic of the heat-transfer model.

All molecular dynamics simulations in this work were performed using LAMMPS (4 Feb 2025). Two Ti–water models were established, as shown in Figure 1. The first model was used for interfacial heat-transfer calculations, in which a water layer was confined between two Ti slabs to form a solid–liquid–solid system (Figure 1a). The simulation domain was 60 Å × 60 Å × 180 Å in the x, y, and z directions, respectively. The confined water region contained 7200 water molecules, corresponding to a density of 0.998 g/cm^3^. The Ti slabs were constructed from hexagonal close-packed (HCP)-Ti with lattice constants of a = 2.944 Å and c = 4.674 Å. The Ti slabs exposed the Ti(0001) surface to contact with water.

**Figure 2:**
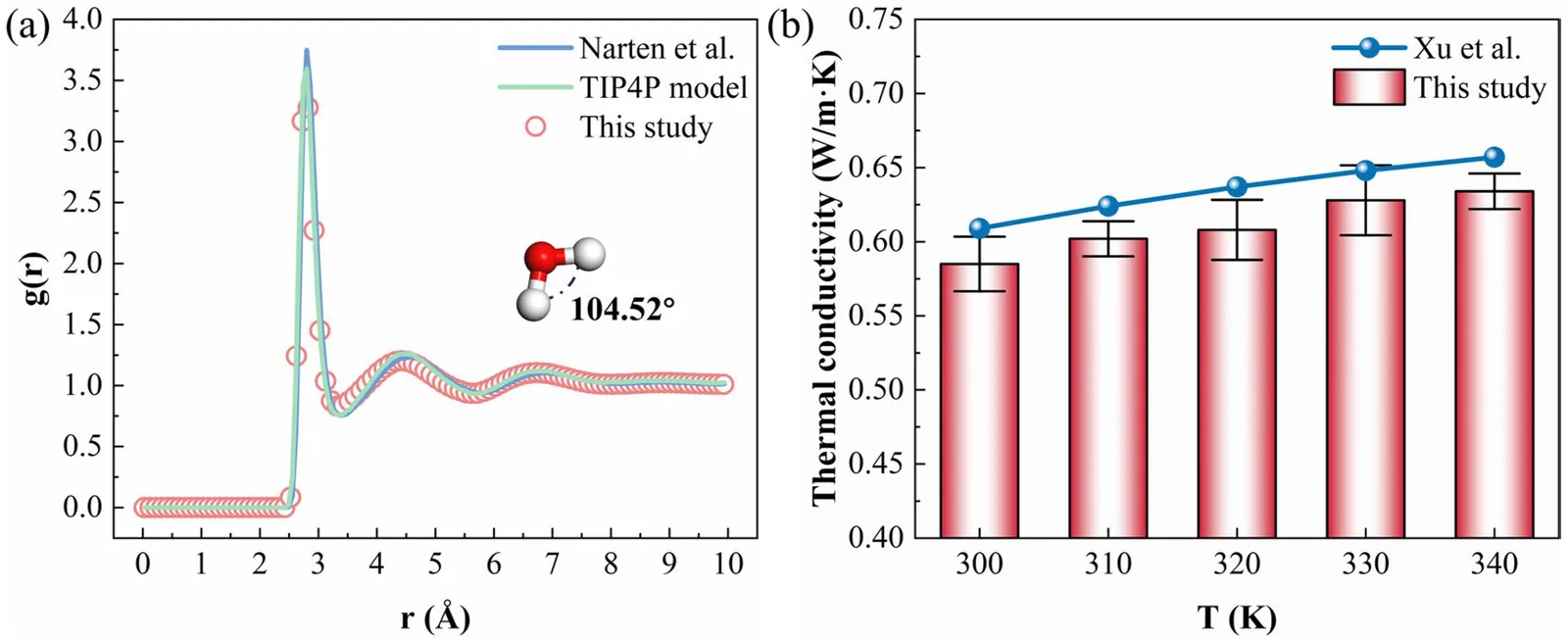
Validation of the water model. (**a**) O-O RDF compared with the experimental data of Narten et al. [33] and the previously reported TIP4P/2005 results [34] at 300 K. (**b**) Thermal conductivity of water compared with the experimental data of Xu et al. [35] at different temperatures under an interaction strength of 0.005 eV.

For the wettability calculation, a separate droplet model was constructed by placing a spherical water cluster on a Ti(0001) slab (Figure 1b). The Ti slab in this model was 120 Å × 120 Å × 20 Å, and the initial water droplet had a radius of 20 Å and contained 1200 water molecules. The TIP4P/2005 model was used for water molecules. Metallic bonding in the Ti substrate was described using a Finnis–Sinclair-type embedded-atom method (EAM/FS) potential. The nonbonded interaction between Ti and water was represented by a Lennard-Jones potential, in which the Ti-O interaction strength was systematically varied. The detailed forms of the interaction potentials and the corresponding parameters are provided in Method S1 and Table S1 in the Supporting Information, respectively. The reliability of the water and Ti models was validated before the simulations. For water, the simulated O-O RDF was compared with both experimental data [33] and previously reported TIP4P/2005 results [34] (Figure 2a). The calculated thermal conductivity was also compared with experimental values at different temperatures [35] (Figure 2b). The deviation in thermal conductivity remained below 10% over the examined temperature range. These results show that the TIP4P/2005 model gives a reasonable description of both the liquid structure and the thermal-transport properties of water. For the Ti substrate, the simulated Ti-Ti RDF was compared with the data reported by Hazarika et al. [36]. The peak positions are in good agreement with the reference result (Figure S1), indicating that the constructed HCP-Ti substrate has a reliable atomic structure.

### 2.2. Ti-O Interaction Modulation

To control the wettability of the Ti surface, only the Ti-O interaction strength was varied in this work. The model construction and force-field settings described above were kept unchanged for all cases. Thus, the wettability and interfacial heat-transfer calculations were compared under the same model framework. The Ti-O interaction strength was first varied over 0.004 eV−0.028 eV in the droplet simulations. For each droplet system, energy minimization was performed to remove unfavorable initial configurations. The system was then relaxed in the NVT ensemble at 300 K for 1 ns and subsequently simulated in the NVE ensemble for 10 ns to obtain the equilibrated droplet morphology. All contact angles were measured at 300 K. The contact angle was determined from the equilibrated droplet trajectory by fitting the gas–liquid interface near the three-phase contact region, as shown in Figure S2. The detailed procedures for the statistical averaging and circle fitting are provided in Method S2.

The initial droplet size was also checked before the wettability analysis. Water droplets with different initial radii were compared, and the contact angle became nearly stable when the radius reached 20 Å (Figure S3). Therefore, the droplet model with an initial radius of 20 Å was used in this work. The work of adhesion between the Ti surface and water was calculated using the phase-separation method and the Young–Dupré relation, as described in Method S3 and Method S4. The contact-angle results were then used to define the wetting cases for the heat-transfer calculations. Interfacial heat-transfer calculations were performed at Ti-O interaction strengths of 0.005 eV, 0.010 eV, 0.015 eV, 0.020 eV, and 0.025 eV. These cases cover the transition from weak to strong wetting observed in the droplet simulations. For each interaction strength, the same Ti-O parameter was used in both the droplet model and the solid–liquid–solid heat-transfer model. The complete sets of Ti-O interaction strengths used in the droplet model and in the heat-transfer model are listed in Table S2.

### 2.3. Interfacial Heat-Transfer Calculation

The heat-transfer model shown in Figure 1c was used for the NEMD calculations. A timestep of 1 fs was used, with a cutoff of 10 Å for the nonbonded interactions and the particle–particle particle–mesh (PPPM) method for the long-range electrostatics. Before imposing a temperature gradient, each system was equilibrated to obtain a stable initial state. The equilibration was confirmed by monitoring the pressure and stress components of the Ti substrate (Figure S4) and the density and pressure of the confined water (Figure S5), which verified that both the Ti structure and the water remained stable. In the NEMD simulations, periodic boundary conditions were applied in the x and y directions. The Ti atoms at both ends of the simulation domain along the z direction were fixed to maintain the structural stability of the system. Two 4 Å thick regions adjacent to the fixed layers were assigned as the heat source and heat sink, respectively, generating a heat flux across the Ti–water interfaces along the z direction. The steady state of heat transfer was verified from the stable temperatures of the heat source, heat sink, and system (Figure S6). The local temperature was calculated from the atomic kinetic energy in each slab:

(1) $$T=\frac{\sum m_{i}v_{i}^{2}}{3Nk_{B}}$$

where *N* is the number of atoms in the slab; *k_B_* is the Boltzmann constant; *m_i_* and *v_i_* are the mass and velocity of atom i, respectively. The temperature profile was obtained by dividing the simulation domain into slabs along the z direction and averaging the local temperature during the steady-state stage. The steady heat flux was calculated from the energy-exchange rate between the heat source and heat sink:

(2) $$J=\frac{dE}{2A\times dt}$$

where *A* is the interfacial area and d*E*/d*t* is the energy-exchange rate. The interfacial temperature difference was obtained from the temperature jumps at the two Ti–water interfaces, and a representative temperature profile is shown in Figure S7.

(3) $$G=\frac{J}{\Delta T}=\frac{1}{R}$$

where Δ*T* is the temperature jump at the interface and *R* is the ITR. For each Ti-O interaction strength, five independent simulations with different initial velocity seeds were performed, and the reported values were averaged over these runs with the uncertainty estimated from their standard deviation. The reliability of the NEMD results was further verified by a series of tests on the system size, sampling time, and linear response. The ITC was confirmed to be independent of the water-film thickness, the cross-sectional area, and the Ti-slab thickness, to be converged with respect to the sampling duration, and to lie in the linear-response regime with respect to the imposed temperature difference (Figures S8–S15 and Tables S3–S5). The complete equilibration and heat-transfer calculation procedures are described in Method S5.

### 2.4. Interfacial Structure and Dynamics

The solid–liquid interfacial region in the heat-transfer model was identified from the atomic density profile along the z direction. On the solid side, the interface was represented by the outermost Ti atoms in direct contact with water. On the liquid side, the first and second water layers near the Ti surface were selected according to the first two peaks in the water-density profile, as shown in Figure S16. These interfacial Ti atoms and water layers were used for the following structural and vibrational analyses. The short-range correlation at the Ti–water interface was characterized using the RDF, and the Ti-O RDF was calculated between surface Ti atoms and water oxygen atoms to describe the local coordination of interfacial water around the Ti atoms. The calculation details of the RDF are provided in Method S6.

The interfacial water structure was further characterized by water orientation distribution, density depletion length, and *F*_4_ order parameter. The water orientation distribution describes the arrangement of water dipoles near the Ti surface (Method S7). The density depletion length measures the separation between the Ti surface and the adjacent water layer (Method S8). The *F*_4_ order parameter reflects the local ordering of the hydrogen-bond network in the interfacial water region (Method S9). The vibrational coupling at the Ti–water interface was analyzed using the VDOS of interfacial Ti atoms and interfacial water molecules. The VDOS overlap was used to evaluate the spectral matching between the solid and liquid sides. For water molecules, the low-frequency diffusive contribution to the VDOS was separated to obtain a clean vibrational spectrum before calculating the spectral overlap. The VDOS calculation, spectral-overlap analysis, and water-VDOS decomposition are described in Method S10 and Figure S17. Phonon heat capacity, phonon lifetime, and envelope decay time were also calculated to characterize the interfacial vibrational energy-transfer behavior, as described in Method S11 and Method S12.

## 3. Results and Discussion

### 3.1. Interfacial Wettability and Adhesion

**Figure 3:**
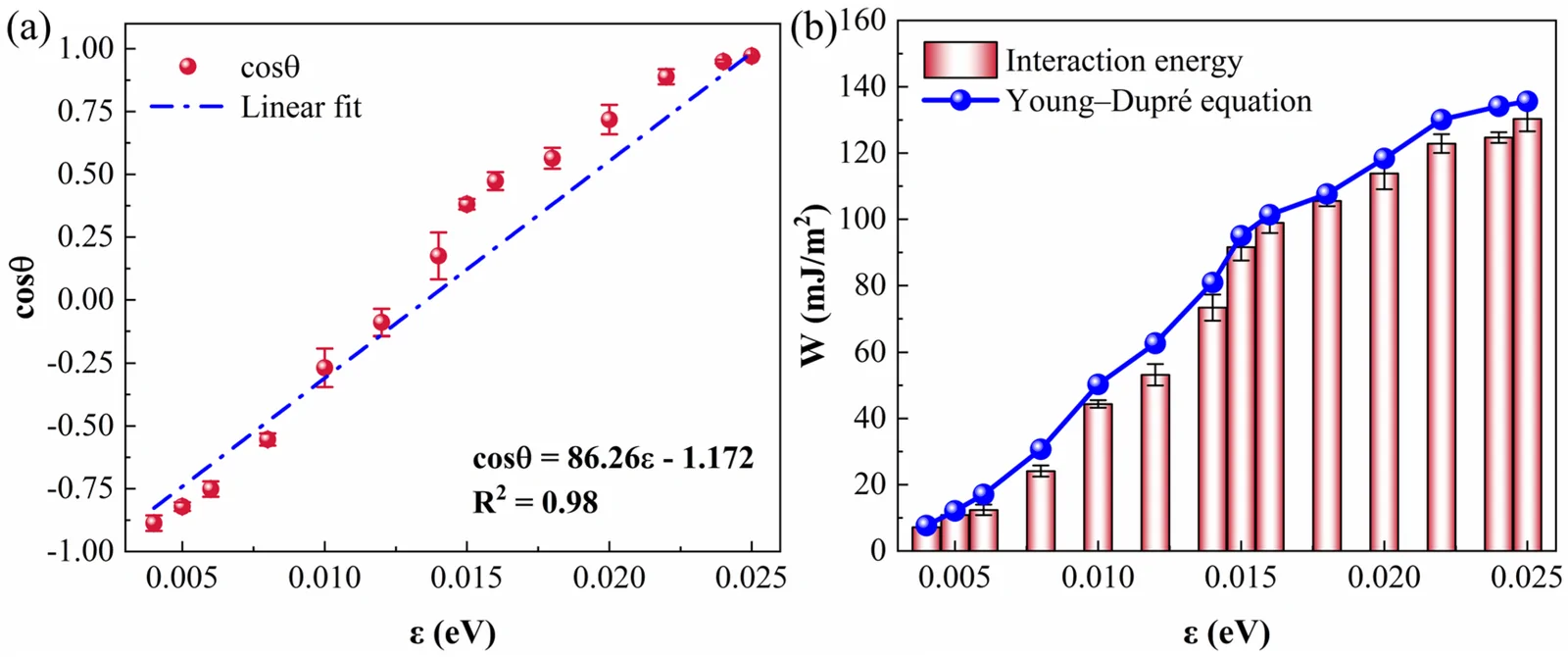
(**a**) Cosine of the water contact angle and (**b**) interaction energy and work of adhesion versus Ti-O interaction strength.

The effect of the Ti-O interaction strength on the interfacial wettability was first examined through the variation of the water contact angle. As shown in Figure 3a, as the interaction strength increased from 0.004 to 0.028 eV, the contact angle decreased continuously from 153° to complete wetting (θ < 5°). This variation is directly visible in the side-view snapshots of the equilibrated droplet (Figure S18), where the droplet gradually spreads from a nearly hemispherical shape in the hydrophobic state into a thin film covering the surface as the interaction strength increases. The same trend is also reflected in the density distribution of the water droplet (Figure S19). At the strongest interactions (ε = 0.026 and 0.028 eV), the water no longer forms a discernible droplet but instead spreads into a thin adsorbed film covering the Ti surface, corresponding to the complete-wetting regime. In this regime, a circle fit to the liquid–vapor profile no longer yields a well-defined Young contact angle, and the corresponding cases were therefore reported as complete wetting (θ < 5°) and excluded from the cos θ-ε fit. An approximately linear relationship is observed between the cosine of the contact angle and the interaction strength, with the fitted equation cos θ = 86.26ε − 1.172. The linear fit is used here as an approximate description over the partial-wetting range. The two fully wetted cases (ε = 0.026 and 0.028, θ < 5°) were excluded from the fit, since the droplet has spread into a film and the contact angle is no longer well defined in this regime. A stronger interaction strength enhances the attraction between Ti and water molecules and lowers the solid–liquid interfacial free energy. According to the balance of interfacial tensions described by the Young equation, this reduction in interfacial free energy directly corresponds to a smaller contact angle. Because the Young equation relates the interfacial free energy to cos θ rather than to θ itself, cos θ increases approximately linearly with the interaction strength. The fitted equation gives cos θ = 0, corresponding to a contact angle of 90°, at an interaction strength of about 0.0136 eV, which should be regarded as an estimate given the approximate linearity of the fit, corresponding to the transition of the interface from hydrophobic to hydrophilic. It should also be noted that the contact angles obtained from nanoscale droplets are subject to line-tension and finite-size effects. According to the modified Young equation, $\cos\theta =\cos\theta_{\infty }-\frac{\tau_{\mathrm{line}}}{\gamma_{lv}\times R}$, where $\tau_{\mathrm{line}}$ is the line tension and *R* is the droplet base radius, the measured contact angle deviates from its macroscopic value $\theta_{\infty }$ by a term inversely proportional to *R*. As shown in Figure S3, the contact angle becomes essentially independent of the initial radius beyond 20 Å, indicating that this finite-size effect is well controlled at the droplet size adopted in this work, so that the reported contact angles reliably characterize the surface wettability. Similarly, Chen et al. [37] also observed that the wettability increased with the solid–liquid interaction strength and eventually approached complete wetting.

To quantify the interfacial binding strength, the interaction energy and the work of adhesion were further analyzed as functions of the interaction strength (Figure 3b). The interaction energy obtained from the phase-separation method (Method S3) and the work of adhesion obtained from the Young–Dupré equation (Method S4) both increased monotonically with the interaction strength. It should be noted that these two quantities are not strictly equivalent and are obtained from different geometries. The phase-separation interaction energy is computed from the potential-energy difference between the coupled and separated systems, normalized by the base area of the droplet obtained from the circle fit (Method S3), whereas the Young–Dupré work of adhesion is a free-energy quantity derived from the contact angle (Method S4). Because the droplet base area used in the phase-separation method itself depends on the fitted contact angle, the closeness of the two curves in Figure 3b partly reflects this shared geometric input rather than an independent mutual validation. The phase-separation interaction energy increased from 7.21 ± 0.16 mJ/m^2^ at 0.004 eV to 130.29 ± 3.76 mJ/m^2^ at 0.025 eV, and the Young–Dupré work of adhesion increased correspondingly, remaining below the work of cohesion of water. The work of adhesion directly reflects the strength of the solid–liquid interfacial binding. A larger interaction strength leads to a stronger attraction of the Ti surface to the interfacial water molecules, a tighter interfacial binding, and a higher energy required to separate the interface, so that the work of adhesion increases accordingly. This trend corresponds to the decrease in the contact angle in Figure 3a. At the hydrophobic-to-hydrophilic transition point at an interaction strength of about 0.0136 eV, the Young–Dupré relation gives a work of adhesion equal to the liquid–vapor surface tension of water, 68.4 mJ/m^2^ [38], which is exactly half of the work of cohesion of water and corresponds to the balance between the interfacial binding and the cohesion of water itself. As the interaction strength approaches complete wetting, the Young–Dupré work of adhesion approaches the work of cohesion of water, 136.8 mJ/m^2^, calculated from the surface tension of this model. This indicates that the solid–liquid interfacial binding strength has approached the cohesion strength of water, and the energy difference between the solid–liquid adhesion and the liquid cohesion nearly vanishes, so that the water droplet tends to spread completely on the Ti surface.

### 3.2. Interfacial Thermal Transport

**Figure 4:**
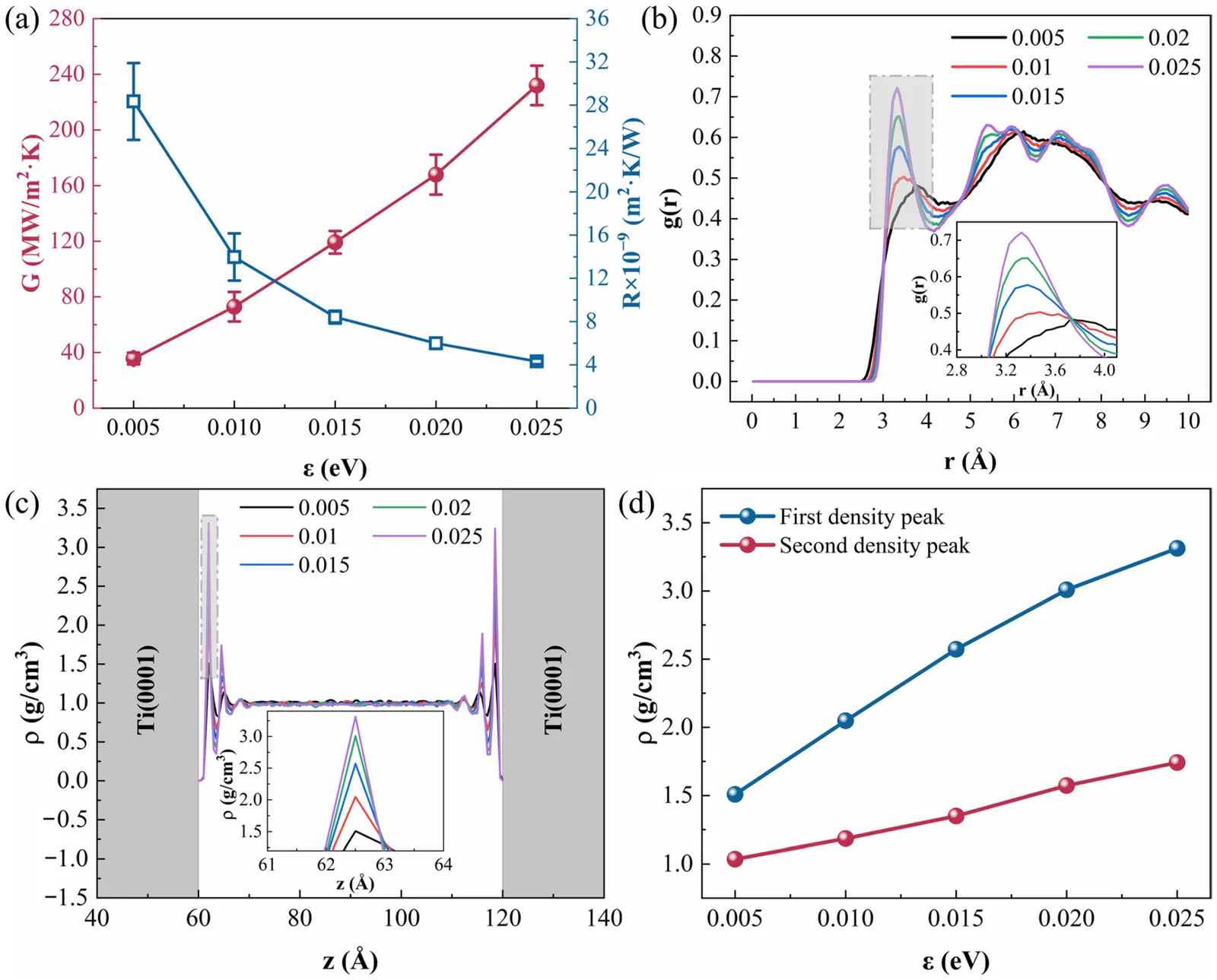
Interfacial heat transfer under different Ti-O interaction strengths. (**a**) ITC and ITR. (**b**) RDF. (**c**) Water-density distribution along the z direction. (**d**) First and second density peaks.

The ITC and ITR under different Ti-O interaction strengths were calculated to examine the effect of the interfacial wettability on the heat transfer between the solid and the liquid. A linear time dependence of the heat-source and heat-sink energies was observed at all interaction strengths (Figure S20), confirming that a steady heat flux had been established. Based on these results, the steady-state heat flux and the interfacial temperature difference yielded the ITC and ITR (Figure 4a). The ITC rose monotonically from 35.71 ± 4.26 to 231.97 ± 14.17 MW/m^2^·K with increasing interaction strength, accompanied by a corresponding reduction in the ITR. The ITC values obtained here, ranging from about 36 to 232 MW/m^2^·K, are comparable in magnitude to the interfacial thermal conductances measured for metal–water interfaces, such as the 50 to 180 MW/m^2^·K reported for Au–water interfaces from hydrophobic to hydrophilic conditions [39]. The increase in ITC was non-uniform, with the increment between adjacent strengths gradually expanding from 37.2 to 64.1 MW/m^2^·K, indicating that the sensitivity of the ITC to the interaction strength increases as the coupling strengthens. The increase in interaction strength corresponds to the transition of the Ti surface from hydrophobic to hydrophilic, which indicates that an interface with better wettability exhibits stronger heat-transfer capability, and that enhancing the solid–liquid coupling effectively improves the cross-interface heat transport. This is further supported by the interfacial interaction energy. The interfacial interaction energy of the solid–liquid–solid model rose from 15.75 ± 1.37 to 120.19 ± 2.93 mJ/m^2^ (Figure S21). This continuous strengthening of the solid–liquid binding provides a stronger coupling basis for cross-interface heat transfer. To clarify the microscopic mechanism of this enhancement, the Ti-O RDF and the water-density distribution were further analyzed (Figure 4b,c). The first nearest-neighbor peak of the Ti-O RDF is located at 3.3 Å, and its height increases significantly with increasing interaction strength. This indicates that the oxygen atoms of the interfacial water molecules aggregate more closely around the Ti atoms and the solid–liquid short-range correlation is enhanced, providing a more efficient atomic-scale channel for cross-interface heat transport. This enhanced short-range correlation is also reflected in the density distribution of interfacial water. Near the Ti(0001) surfaces on both sides, the water density exhibits pronounced oscillatory layering, forming two distinct density peaks, the first and the second density peaks. As the interaction strength increases, the density peak adjacent to the Ti surface rises markedly, indicating that the constraint of the solid-wall potential field on the interfacial water is enhanced, and the interfacial water molecules are more strongly adsorbed onto the surface with a more ordered near-wall arrangement.

As shown in Figure 4d, the first density peak increases from 1.51 to 3.31 g/cm^3^, and the second density peak increases from 1.03 to 1.74 g/cm^3^, with the increase in the first density peak being clearly larger than that in the second. This difference indicates that the increase in interaction strength has the most pronounced effect on WL1, which is adjacent to the solid, whereas the second water layer (WL2), being farther from the surface and less modulated by the wall potential field, shows a relatively gentle density change. WL1 plays a key role in the solid–liquid heat-transfer process. Therefore, the increase in interaction strength strengthens the vibrational coupling at the solid–liquid interface by enhancing the Ti-O short-range correlation and increasing the density of WL1, which in turn drives the increase in ITC. It should be noted that the increase in ITC is clearly larger than that in WL1 density, and the two are not linearly correlated, which implies that the enhancement of interfacial heat transfer originates not only from the increase in interfacial water density but also from the improvement in the efficiency of solid–liquid vibrational coupling.

**Figure 5:**
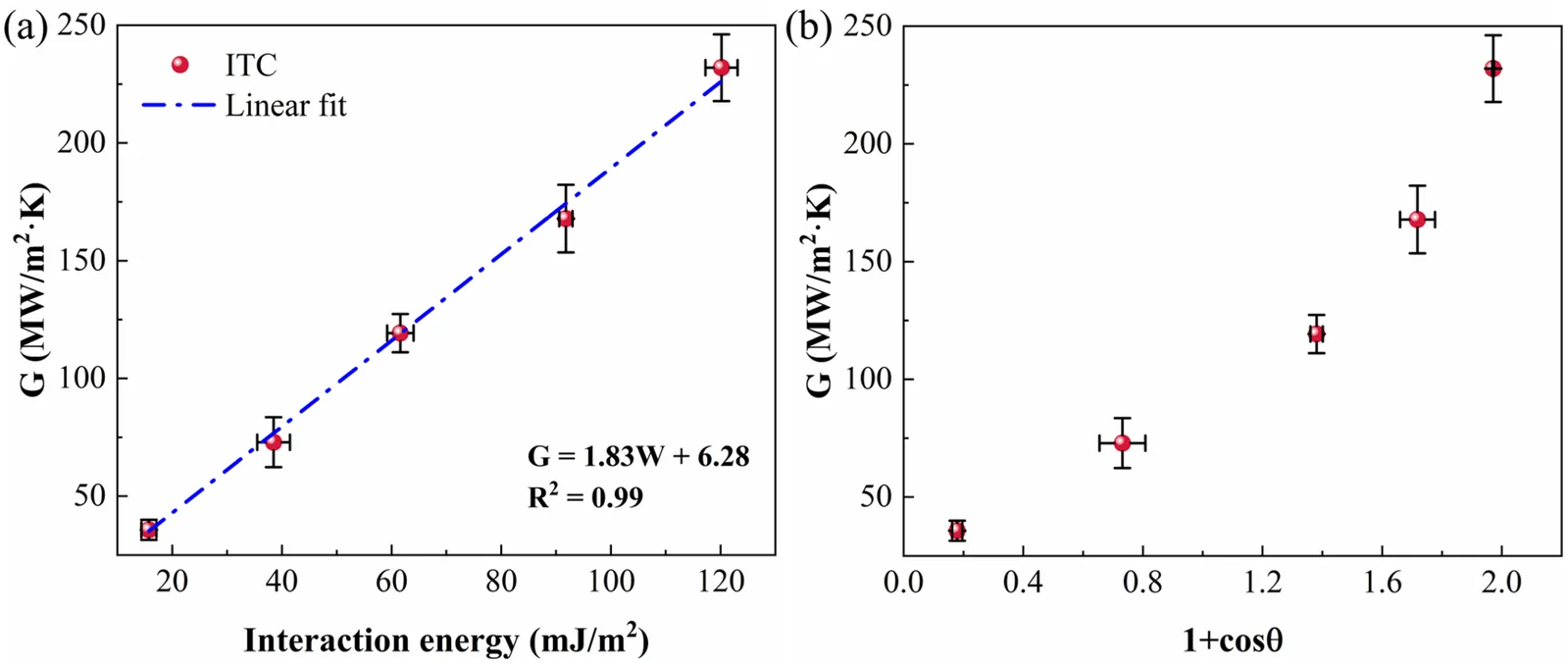
Scaling behavior of the ITC with the interfacial interaction energy and (1 + cos θ). (**a**) ITC against the interfacial interaction energy; (**b**) ITC against (1 + cos θ).

To further examine the physical origin of the ITC enhancement, we tested the present data against the classical scaling relations (Figure 5). As shown in Figure 5a, the ITC increases almost perfectly linearly with the interfacial interaction energy obtained from the phase-separation method. In contrast, the ITC does not vary linearly with (1 + cos θ) (Figure 5b). The data deviate clearly from a straight line, and the deviation is most pronounced in the strong-wetting regime, where (1 + cos θ) approaches its upper limit of 2 while the ITC continues to increase. This indicates that the classical wettability-based scaling captures only the overall trend and cannot quantitatively reproduce the ITC near complete wetting, whereas the interfacial interaction energy remains effective in characterizing the ITC across the entire range. The contrast between Figure 5a and Figure 5b reflects that the phase-separation interaction energy and the Young–Dupré work of adhesion are not equivalent measures of adhesion. Over the interaction-strength range studied here, the three adhesion-related quantities increase at different rates, as shown in Figure S22 and listed in Table S6. The solid–liquid–solid interaction energy varies almost linearly with the interaction strength, whereas the droplet interaction energy and the Young–Dupré work of adhesion rise more steeply in the intermediate range and more slowly toward the strongest interactions studied, so that their ratio to the solid–liquid–solid interaction energy changes systematically across the range. This difference in curvature, rather than any saturation, distinguishes the two measures over the range studied, and is consistent with the linear relation in Figure 5a and the nonlinear one in Figure 5b. We report this divergence as an observation. Consequently, the ITC scales linearly with the phase-separation interaction energy but not with (1 + cos θ), and the defensible conclusion is that the ITC is controlled by the interfacial coupling strength as measured by the phase-separation interaction energy, rather than by the wettability-based (1 + cos θ) scaling.

### 3.3. Structure of Interfacial Water

**Figure 6:**
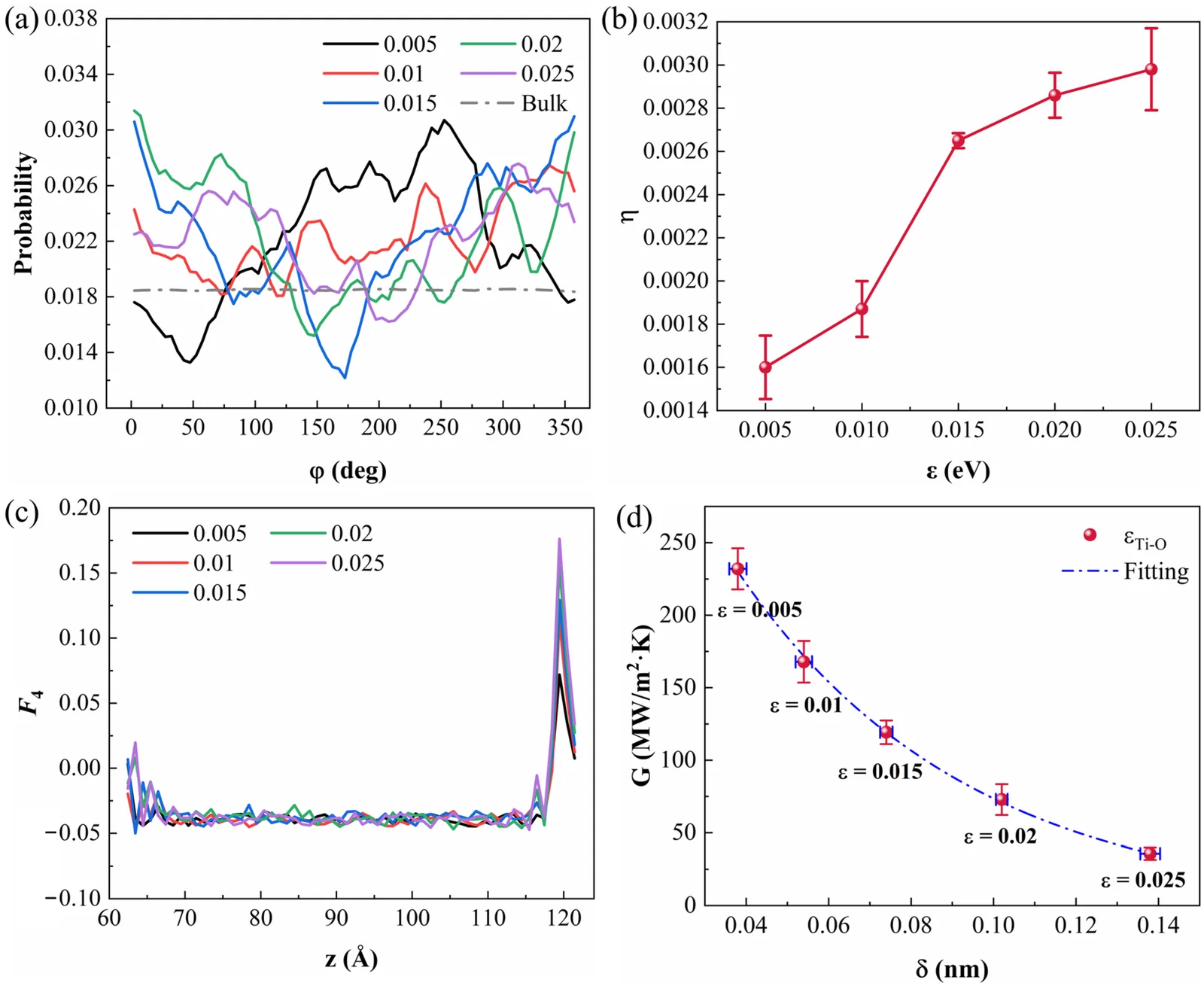
Structure of interfacial water under different Ti-O interaction strengths. (**a**) Probability distribution of the orientation angle for WL1. (**b**) Quantitative description of the fluctuations in the probability distribution. (**c**) *F*_4_ order parameter of the interfacial water. (**d**) ITC versus density depletion length.

The enhancement of interfacial heat transfer has been attributed above to the densification of interfacial water, while its orientation and structure also change with the interaction strength and jointly affect the cross-interface heat transfer. These changes in the interfacial water are first reflected in its molecular orientation. Under the potential field of the solid, the interfacial water deviates from the random orientation of the bulk and exhibits in-plane orientational ordering. As shown in Figure 6a, the orientation angle distribution of bulk water is nearly uniform, whereas that of WL1 shows pronounced oscillations whose amplitude increases with the interaction strength. This ordering is quantified by the fluctuation of the orientation angle distribution (Figure 6b), where the order parameter increases from 0.0016 ± 0.00014 to 0.00298 ± 0.00018, with the most significant rise occurring as the interaction strength increases from 0.01 to 0.015. A stronger attraction from the solid surface suppresses the thermal disturbance of the orientation, so that the interfacial water maintains a specific orientation more stably and the orientational ordering increases. This ordering is usually accompanied by the reorganization of the hydrogen-bond network, which is more directly reflected in the four-body structure. Figure 6c presents the distribution of the *F*_4_ parameter along the z direction. In the bulk region, the *F*_4_ parameter remains stable at about −0.04, consistent with the typical value of bulk water, whereas at the interface it becomes positive, showing that the hydrogen-bond network of the interfacial water is reconstructed and the four-body arrangement tends to be ordered. As the interaction strength increases, the interfacial *F*_4_ parameter rises continuously from 0.072 to 0.176 and increases more rapidly in the low-strength region, consistent with the trend of the orientational ordering. The orientational and four-body ordering thus strengthen together, and the interfacial water becomes increasingly ordered with the interaction strength.

The ordering of interfacial water is accompanied by a denser near-wall structure, which can be characterized by the density depletion length. Figure 6d presents the variation in ITC with the density depletion length. As the interaction strength increases, the density depletion length decreases from 0.138 ± 0.0024 to 0.038 ± 0.0021 nm, while the ITC increases from 35.71 ± 4.26 to 231.97 ± 14.17 MW/m^2^·K. The ITC increases exponentially with decreasing density depletion length, following G = 461.03 exp (− δ/0.055) − 2.58, so that a smaller density depletion length corresponds to a faster increase in ITC, which is particularly evident in the dense interfacial region. Denser interfacial water brings the solid and the liquid into closer contact and allows more sufficient vibrational coupling, so that heat is transferred across the interface more easily. Taken together, the enhanced orientational and four-body ordering, the increased near-wall density, and the reduced density depletion length consistently show that a stronger interaction strength drives the interfacial water into a denser, more ordered, and more tightly bound near-wall structure. This tight binding brings the solid and the liquid into close and stable contact and strengthens the solid–liquid vibrational coupling, thereby promoting the increase in ITC.

### 3.4. VDOS at the Solid–Liquid Interface

**Figure 7:**
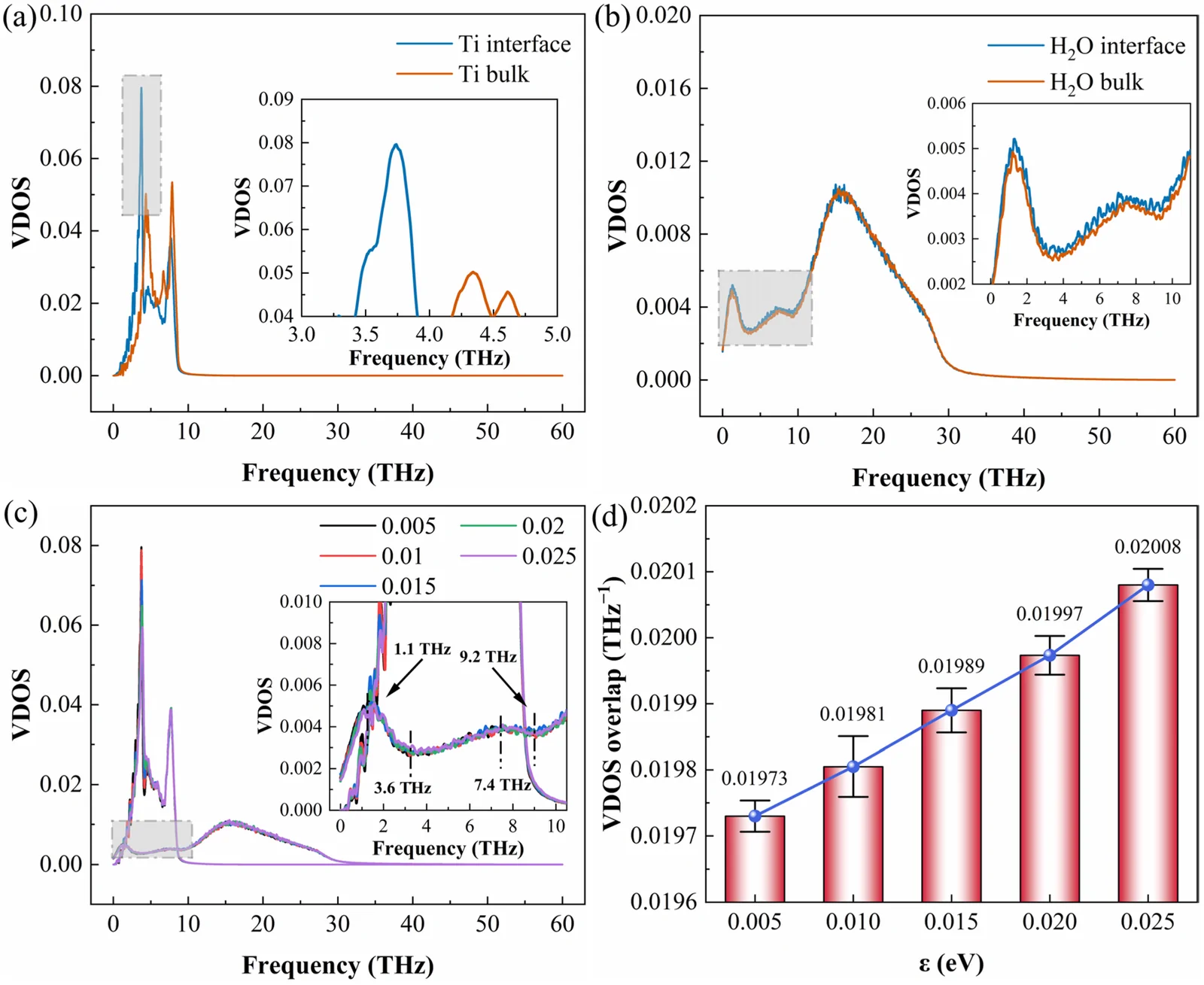
VDOS of the interfacial and bulk (**a**) Ti and (**b**) water. (**c**) VDOS under different interaction strengths. (**d**) Out-of-plane (z) VDOS overlap at the solid–liquid interface.

Interfacial heat transfer is essentially the transfer of phonons across the solid–liquid interface, so the matching of the solid and liquid vibrational spectra governs the cross-interface heat transfer. Figure 7a,b present the VDOS of Ti and water at the interface and in the bulk. The vibrations of Ti are mainly distributed below 10 THz, and the main peak of the interfacial Ti shifts toward lower frequency and is enhanced relative to the bulk, because the interaction with water weakens the constraint on the interfacial Ti atoms. The vibrations of water span a wider range with a main peak at 15 THz, and the interfacial water shows a slightly higher VDOS in the low-frequency region, which is the most relevant to the cross-interface phonon transfer.

As shown in Figure 7c, the spectra for the five interaction strengths nearly coincide, indicating that the overall spectral shape is insensitive to the interaction strength, with discernible differences appearing mainly at the main peak of the interfacial Ti (Figure S23), which gradually decreases, broadens, and shifts to a higher frequency as the interaction strengthens. Since the cross-interface heat flux is transported mainly along the direction normal to the interface, the spectral matching was evaluated using the out-of-plane (z) VDOS. The spectral overlap is defined as the frequency integral of the product of the solid and liquid VDOS, normalized by the product of their individual integrals (Method S10), so that it reflects only the matching between the shapes of the two spectra. Figure 7d shows that the out-of-plane overlap increases monotonically from 0.01973 ± 0.000025 to 0.02008 ± 0.000012 THz^−1^, by only about 2%, over the same range in which the ITC increases by a factor of 6.5. This pronounced difference in magnitude shows that the spectral overlap is not the dominant factor controlling the interfacial heat transfer.

A directional and scaling analysis further supports this conclusion. When the VDOS is decomposed into out-of-plane (z) and in-plane (xy) components (Figure S24), the out-of-plane overlap increases by about 1.8% while the in-plane overlap increases by only about 0.5% (Figure S25). The out-of-plane overlap is thus more sensitive to the interaction strength, consistent with the unidirectional heat flux, yet even this most relevant component varies by less than 2%, showing that the weak variation of the overlap is intrinsic rather than a consequence of mixing the in-plane modes. This weak variation is further quantified by power-law fits of the form y ∝ ε^n^ over the interaction-strength range of 0.005 to 0.025 eV. The ITC scales with the interaction strength with an exponent of about 1.16, close to that of the interaction energy (about 1.26), whereas the spectral overlap scales with an exponent of only about 0.01. It should be noted that the spectral overlap is a bounded quantity of different dimensions from the interaction energy, so its small exponent reflects only that a bounded quantity varies little and does not by itself establish causality. Since the interaction strength is the only variable in this work, all interfacial quantities necessarily correlate with the coupling strength, and we therefore do not claim the coupling strength as an independent controlling factor beyond this parametrization. What can be robustly concluded is the negative statement that the spectral overlap, varying by less than 2% while the ITC changes by a factor of 6.5, is not the dominant factor controlling the interfacial heat transfer. The interfacial densification, the increased near-wall ordering, and the accelerated interfacial vibrational energy exchange are concurrent consequences of the enhanced interfacial coupling that together promote the increase in ITC.

**Figure 8:**
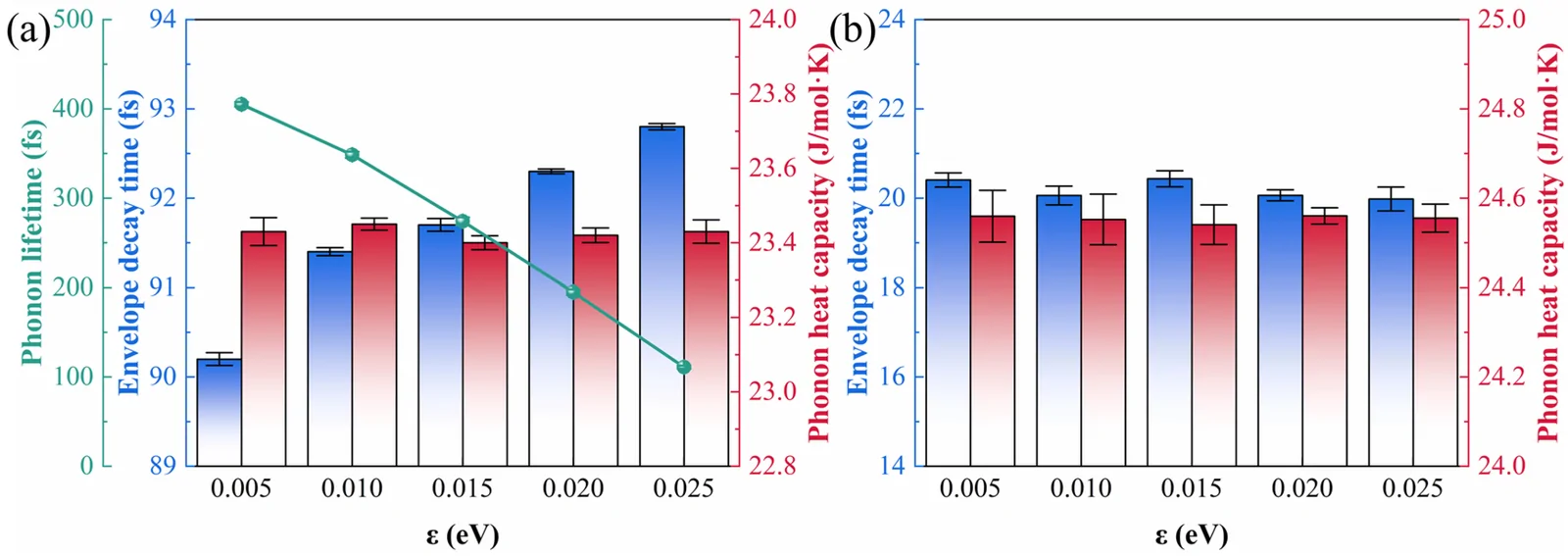
Envelope decay time, phonon lifetime, and phonon heat capacity under different Ti-O interaction strengths. (**a**) Interfacial Ti. (**b**) Interfacial water.

To gain further insight into the phonon transfer efficiency, the envelope decay time, phonon lifetime, and phonon heat capacity were evaluated for both the interfacial Ti and the interfacial water (Figure 8a,b). For both species, the phonon heat capacity is essentially independent of the interaction strength, remaining around 23.4 J/mol·K for the Ti and about 20.2 J/mol·K for the water, both below the classical upper limit of 3R. The enhancement of interfacial heat transfer therefore does not originate from a change in the heat capacity, but should be attributed to the improvement in the phonon transfer efficiency. The phonon lifetime of the interfacial Ti, obtained from the Lorentzian linewidth of its well-resolved main peak, decreases significantly with increasing interaction strength, from 405 ± 2.7 to 111 ± 1.8 fs. For the interfacial water, the low-frequency band is overdamped and does not form a spectrally resolvable peak, so its characteristic time is taken from the envelope decay time $\tau_{\mathrm{env}}$ rather than from a linewidth-based lifetime. The shortening of the Ti phonon lifetime means that the interfacial Ti vibrations exchange energy with the water modes within a shorter time, which corresponds to a stronger solid–liquid coupling, since interfacial heat transfer depends on the coupling of the vibrations on both sides rather than on the long-range transport in bulk conduction. This trend is consistent with the broadening of the main peak of the interfacial Ti (Figure S23). The envelope decay time characterizes the overall decay behavior of the interfacial atomic vibrations. For the interfacial Ti, it increases slowly from 90.2 ± 0.07 to 92.8 ± 0.04 fs with interaction strength, showing that the interfacial atoms vibrate more regularly and persistently under the stronger constraint. For the interfacial water, the envelope decay time fluctuates between 24 and 25 fs without a clear trend. With the heat capacity essentially unchanged, the stronger interaction thus improves the cross-interface energy-transfer efficiency mainly by shortening the phonon lifetime of the interfacial Ti.

## 4. Conclusions

In this work, molecular dynamics simulations were used to study the effect of the Ti-O interaction strength on heat transfer across Ti–water interfaces, and the regulation of ITC was analyzed in terms of interfacial wettability, interfacial water structure, and phonon vibrational properties. As the interaction strength increased from 0.005 to 0.025 eV, the ITC rose from 35.71 ± 4.26 to 231.97 ± 14.17 MW/m^2^·K, and the increment between adjacent strengths tended to increase, showing that the ITC rose progressively with the interaction strength. Over this range, the water contact angle decreased from about 143° to 15°, and the cosine of the contact angle increased approximately linearly with the interaction strength. Across the full droplet series from 0.004 to 0.028 eV, the Ti surface changed from strongly hydrophobic (θ = 153°) to complete wetting (θ < 5°). The work of adhesion increased with the interaction strength and approached the work of cohesion of water near full wetting, indicating that the wettability is determined by the relative strength of the solid–liquid adhesion and the liquid cohesion. The increase in ITC originated from the change in the interfacial water structure. As the interaction strengthened, WL1 became denser and more ordered and the density depletion length decreased, so that the interfacial water became more tightly bound to the surface. The interfacial water thus formed a denser and more ordered near-wall structure, which provided a stronger basis for the solid–liquid vibrational coupling. From the phonon perspective, the spectral overlap increased by only about 2%, from 0.01973 to 0.02008 THz^−1^, while the ITC increased by a factor of 6.5, indicating that the spectral overlap is not the controlling factor of the interfacial heat transfer. Instead, the phonon lifetime of the interfacial Ti shortened markedly from 405 to 111 fs, while the phonon heat capacity remained stable and below the classical limit, showing that the enhancement of the interfacial heat transfer originates from the stronger interfacial coupling rather than from an increase in the spectral overlap. It should be noted that the present model has certain limitations. It considers a clean, unoxidized Ti surface, whereas real Ti forms a TiO_2_ passivation layer in water, so the model serves as a parametric study rather than a direct representation of a specific experimental interface. Moreover, the electron–phonon coupling on the metal side is absent from classical molecular dynamics, and the ITC obtained here represents the phonon–phonon contribution. Despite these simplifications, the parametric study captures the essential physics of how spectral overlap is not the dominant factor in wettability-dependent interfacial heat transfer. Overall, the Ti-O interaction strength regulates heat transfer across Ti–water interfaces through the coordinated changes in interfacial wettability, interfacial water structure, and phonon coupling, and provides a reference for optimizing the heat dissipation of metal–water interfaces through surface wettability.

## Data Availability

The original contributions presented in this study are included in the article. Further inquiries can be directed to the corresponding author.

## Supplementary Materials

The following supporting information can be downloaded at https://www.mdpi.com/article/10.3390/mi17080972/s1. Figure S1: Comparison of the Ti-Ti RDF of the (0001) substrate model with literature data [36]; Figure S2: Determination of the water contact angle. (a) Snapshot of the equilibrated water droplet on the Ti substrate. (b) Number density distribution for contact angle fitting; Figure S3: Contact angles of water droplets with different initial radii; Figure S4: Pressure and stress components of Ti during equilibration; Figure S5: Density and pressure of water during equilibration; Figure S6: Temperatures of the heat source, heat sink, and system at the steady state; Figure S7: Temperature profile along the z direction across the solid-liquid interfaces; Figure S8: Heat-transfer models with different water-film thicknesses; Figure S9: ITC for different water-film thicknesses at a Ti-O interaction strength of ε = 0.015 eV; Figure S10: Heat-transfer models with different cross-sectional dimensions; Figure S11: ITC for different cross-sectional dimensions at a Ti-O interaction strength of ε = 0.015 eV; Figure S12: Heat-transfer models with different Ti-slab thicknesses; Figure S13: ITC for different Ti-slab thicknesses at a Ti-O interaction strength of ε = 0.015 eV; Figure S14: Convergence of the ITC with simulation time at a Ti-O interaction strength of ε = 0.015 eV; Figure S15: ITC under different imposed temperature differences. The mean temperature was fixed at 300 K for all cases, and the Ti-O interaction strength was fixed at ε = 0.015 eV; Figure S16: Density profiles along the z direction used to determine the solid-liquid interfaces; Figure S17: Separation of the diffusive contribution from the total VDOS to obtain the vibrational component. (a) Interfacial water. (b) Bulk water; Figure S18: Side-view snapshots of the equilibrated water droplet on the Ti surface under different Ti-O interaction strengths, from the hydrophobic state to complete wetting; Figure S19: Number density distributions of the water droplet on the Ti surface under different Ti-O interaction strengths; Figure S20: Energy of the heat source and heat sink versus time under different Ti-O interaction strengths; Figure S21: Interaction energy of the heat transfer model versus Ti-O interaction strength; Figure S22: Comparison of the droplet interaction energy, the solid-liquid-solid interaction energy, and the Young–Dupré work of adhesion as functions of the Ti-O interaction strength; Figure S23: VDOS of the interfacial Ti under different Ti-O interaction strengths; Figure S24: Directional decomposition of the VDOS into out-of-plane (z) and in-plane (xy) components at ε = 0.005 eV. (a) Interfacial Ti. (b) Interfacial water; Figure S25: In-plane (xy) VDOS overlap at the solid-liquid interface under different Ti-O interaction strengths; Table S1: LJ parameters for different atomic pairs; Table S2: The Ti-O interaction strengths used in the droplet model and the heat-transfer model; Table S3: Model parameters of the heat-transfer systems with different water-film thicknesses; Table S4: Model parameters of the heat-transfer systems with different cross-sectional dimensions; Table S5: Model parameters of the heat-transfer systems with different Ti-slab thicknesses; Table S6: The droplet interaction energy, the solid-liquid-solid interaction energy, and the Young–Dupré work of adhesion γlv(1 + cos θ) at the five heat-transfer interaction strengths. Refs. [40,41,42,43,44] are cited in Supplementary Material.
