# Supplementary material for: Linking Surface Wettability to Interfacial Thermal Transport at Ti–Water Interfaces: A Molecular Dynamics Study
Source: Micromachines (Basel). 2026 Aug 18;17(8):972. doi: 10.3390/mi17080972 (PMC13515673; doi:10.3390/mi17080972)
Supplement: Supplementary file 1 [file micromachines-17-00972-s001.zip › micromachines-4431219-supplementary.pdf]

Supporting Information

Linking surface wettability to interfacial thermal transport at Ti-water interfaces: A molecular dynamics study

Haoming Huang [a, b], Xi Wang [c], Ming Ma[d], Shan Qing [a, b*], Zhumei Luo [a, b*], Xiaoyan Huang [b], Jing Zhang [b], Xiaohui Zhang [b]

[a] *State Key Laboratory of Complex Nonferrous Metal Resources Clean Utilization, School of Metallurgical and Energy Engineering, Kunming University of Science and Technology, Kunming 650093, China*

[b] *School of Metallurgical and Energy Engineering, Kunming University of Science and Technology, Kunming 650093, China*

[c] *Yunnan Electric Power Experimental Research Institute (Group) Co., Ltd, China*

[d] *School of Nationality Educators, Qinghai Normal University, Xining 810016, China*

*Corresponding author.

Methods

Method S1: Force-field description and interaction potentials

Water molecules were represented using the rigid four-site TIP4P/2005 model. In this model, Lennard-Jones (LJ) interactions act only on the oxygen site. The two hydrogen sites each carry a charge of +0.5564 e, while the negative charge of −1.1128 e is assigned to a massless M site located along the H-O-H angle bisector, 0.1546 Å from the oxygen atom [34]. The O-H bond length and H-O-H angle are 0.9572 Å and 104.52°, respectively. Since the TIP4P/2005 model treats water as a rigid body, the intramolecular bending (~1600 cm^{-1}) and stretching (~3400 cm^{-1}) modes are absent from the water VDOS. As cross-interface heat transport is dominated by low-frequency modes (< 10 THz), the absence of these high-frequency intramolecular modes has a negligible effect on the interfacial thermal conductance. The O-H bonds and H-O-H angle were constrained using the SHAKE algorithm to maintain the rigid water geometry. Long-range electrostatic interactions were treated using the PPPM method with an accuracy of 1×10^{-6}. A cutoff distance of 10 Å was applied to both LJ and Coulombic interactions [11]. The non-bonded interaction between water molecules was calculated using the LJ and Coulombic potentials:

$$\varphi\left(r_{ij}\right)=4\varepsilon\left[\left(\frac{\sigma}{r_{ij}}\right)^{12}-\left(\frac{\sigma}{r_{ij}}\right)^{6}\right]+\frac{q_i q_j}{4\pi\varepsilon_0 r_{ij}} \tag{S1}$$

where ε is the potential well depth; σ is the size parameter; r_{ij} is the distance between atoms i and j; q is the charge; ε_0 is the vacuum dielectric constant. In addition, the Ti-O interaction was described by the LJ potential.

$$\varphi\left(r_{ij}\right)=4\varepsilon_{\text{Ti-O}}\left[\left(\frac{\sigma_{\text{Ti-O}}}{r_{ij}}\right)^{12}-\left(\frac{\sigma_{\text{Ti-O}}}{r_{ij}}\right)^{6}\right] \tag{S2}$$

Finally, the Ti-Ti interactions were described using a Finnis-Sinclair-type embedded-atom method (EAM/FS) to account for the metallic bonding between Ti atoms [40].

Method S2. Contact-angle calculation

The contact angle was obtained from the equilibrated water droplet morphology. For each Ti-O interaction strength, the trajectory from the last 2 ns of the NVE simulation was divided into five equal time intervals. The density distribution of the water droplet was calculated for each interval, and the gas-liquid interfacial density was taken as $0.5(\rho_l + \rho_v)$, where ρ_l and ρ_v are the liquid-phase and vapor-phase densities, respectively. The gas-liquid interfacial profile was fitted with a circle:

$$(x-a)^2 + (z-b)^2 = R^2 \tag{S3}$$

where a and b are the x and z coordinates of the fitted circle center, respectively, and R is the fitted radius. The contact angle was determined from the tangent at the intersection between the fitted circle and the Ti surface:

$$\theta_c = \cos^{-1}\left(\frac{z_s - b}{R}\right) \tag{S4}$$

where z_s is the z coordinate of the solid-liquid contact plane. The contact angle reported for each interaction strength was obtained by averaging the values from the five statistical intervals.

Method S3: Phase-separation method.

The phase-separation method estimates the interfacial interaction energy by comparing the potential energies of the coupled solid-liquid system and the separated subsystems [41].

$$W_{\mathrm{ad}}^{\mathrm{U}} = \frac{E_{\mathrm{Ti}} + E_{\mathrm{water}} - E_{\mathrm{total}}}{A} \tag{S5}$$

where $W_{\mathrm{ad}}^{\mathrm{U}}$ is the interaction energy per unit area obtained from the potential-energy difference; E_{Ti} is the potential energy of Ti; E_{water} is the potential energy of water; E_{total} is the total potential energy; A is the interfacial contact area. Strictly, the work of adhesion is a free-energy difference, $W_{adh} = \Delta U - T\Delta S$, whereas Eq. (S5) accounts only for the potential-energy (enthalpic) contribution ΔU and neglects the entropic term. Since separating the interface restores the orientational and translational freedom of the interfacial water ($\Delta S > 0$), Eq. (S5) provides an upper bound to the true work of

adhesion.

Method S4: Young-Dupré equation.

Based on the Young-Dupré equation, the work of adhesion can be obtained from the contact angle and the liquid-vapor surface tension [42].

$$W_{ad} = \gamma_l \left(1 + \cos\theta\right) \tag{S6}$$

where γ_l is the liquid-vapor surface tension of water; θ is the equilibrium contact angle.

Method S5: Equilibration and heat-transfer calculation.

All molecular dynamics simulations were performed with a time step of 1 fs, and the equations of motion were integrated using the velocity-Verlet algorithm. The long-range electrostatics were computed using the PPPM method, and a slab correction was applied to account for the non-periodicity along the z direction and the net dipole of the confined water normal to the walls. Energy minimization was first performed to remove unfavorable atomic contacts and reduce the initial potential energy of the system. The system was then equilibrated in the NPT ensemble at 300 K and 1 atm for 1 ns, during which periodic boundary conditions were applied in all three directions and the barostat was applied only along the z direction, with the cross-sectional dimensions in x and y kept fixed, so that the confined water was relaxed to the correct density at 1 atm. At this stage the Ti atoms were not yet fixed. After the NPT equilibration, the outermost Ti atomic layers at both ends along the z direction were fixed, the z direction was then treated as non-periodic, and the system was further relaxed in the NVT ensemble at 300 K for 1 ns using a Nosé-Hoover thermostat to obtain a stable initial configuration for the subsequent non-equilibrium simulation. The non-equilibrium heat-transfer simulation was performed in the NVE ensemble, while a Langevin thermostat was applied to the heat source and heat sink regions to maintain them at 330 K and 270 K, respectively, thereby generating a heat flux along the z direction. The system was first run for 1 ns to reach a steady heat-transfer state. The steady state was identified from the approximately linear cumulative energy exchange between the heat source and heat sink and the stable temperature profile along the z direction. After the steady state was

reached, data were collected for another 1 ns for the temperature and heat-flux calculations, and the reported ITC values are based on this 1 ns production sampling. To verify convergence, the production sampling was additionally extended to 10 ns for a representative case, and the ITC obtained from the 10 ns run agreed with the 1 ns value within the statistical uncertainty (Figure S14), confirming that a 1 ns sampling is sufficient to obtain a converged ITC. The simulation domain was divided into slabs along the z direction, and the local temperature of each slab was calculated from the atomic kinetic energy. Fixed atoms were excluded from the temperature calculation. The temperature jump at the Ti-water interface was obtained by linearly extrapolating the temperature profiles of the Ti and water regions to the solid-liquid boundary. The boundary position was determined from the density profile along the z direction. The heat flux was calculated from the cumulative energy added to and removed from the heat source and heat sink by the Langevin thermostat and the interfacial area. The interfacial thermal conductance was then obtained from the steady heat flux and the interfacial temperature jump. For each Ti-O interaction strength, five independent simulations were performed with different initial random velocity seeds. The reported values were obtained by averaging the independent results, and the uncertainty was estimated as the standard deviation over the five independent simulations.

Method S6: Radial distribution function.

The RDF describes the local atomic density variation around a reference atom and characterizes interfacial coordination at solid-liquid interfaces [43].

$$g(r) = \frac{V}{n_a n_b} \left\langle \sum_{i=1}^{n_a} \frac{n_{ib}(r, \Delta r)}{4\pi r^2 \Delta r} \right\rangle \tag{S7}$$

where V is the volume; n_a and n_b are the number of particles; r is the radial separation; Δr is the shell width; $n_{ib}(r, \Delta r)$ is the number of b particles within a radius from r to $r+\Delta r$ around a particle; the bracket $\langle \; \rangle$ denote an equilibrium ensemble average.

Method S7: Dipole orientation of interfacial water.

To characterize the orientational structure of interfacial water, the dipole

orientations of water molecules in the first water layer (WL1) were analyzed under different Ti-O interaction strengths. The angle (φ)was defined as the angle between the projection of the water dipole vector onto the x-y plane and the x-axis. The probability distribution of φ was then calculated to evaluate the in-plane orientational ordering of interfacial water molecules [44].

$$\eta = \sqrt{\frac{1}{N}\sum_{i=1}^{N}\left(p_i - \langle p_i \rangle\right)^2} \tag{S8}$$

where N is the total number of intervals; p_i is the probability of the angle φ falling into the ith interval; $\langle p_i \rangle$ is the average value of the probability.

Method S8: Density depletion length.

The density depletion length was used to quantify the effective thickness of the density-depleted interfacial water layer relative to bulk water, thereby characterizing the interfacial water density structure under different wettability conditions [18].

$$\delta = \int_0^{\infty}\left[1 - \frac{\rho_S(z)}{\rho_S^b} - \frac{\rho_L(z)}{\rho_L^b}\right] dz \tag{S9}$$

where $\rho_S(z)$ and $\rho_L(z)$ are the solid and liquid densities along the z-axis; ρ_S^b and ρ_L^b are the bulk solid and liquid densities.

Method S9: Four-body structure order parameter.

The four-body structure order parameter (F_4) order parameter is widely employed in MD simulations to quantify the structural arrangement of interfacial water molecules. The F_4 parameter is defined based on the torsional angle formed by the outermost H and O atoms of two neighboring water molecules, and is given by:

$$F_4 = \frac{1}{n}\sum \cos 3\Phi \tag{S10}$$

where Φ is the H-O⋯O-H torsional angle between two adjacent water molecules, which is only considered when the oxygen-oxygen distance is within 3.5 Å; n is the total number of selected water molecule pairs. For bulk water, the F_4 order parameter

is -0.04. Although the F_4 order parameter was originally introduced to identify clathrate hydrates, it is a general measure of the hydrogen-bond dihedral configuration between neighboring water molecules and takes distinct values for different water structures, so it is sensitive to the ordering of water. Here it is used to compare the interfacial water with the bulk water and to characterize the enhancement of the interfacial water ordering with increasing interaction strength, rather than to identify a specific crystalline or hydrate phase.

Method S10: VDOS calculation and separation of diffusive and vibrational contributions

The vibrational density of states (VDOS) characterizes the vibrational modes over frequency, with spectral overlap reflecting phonon coupling and heat transfer pathways. The VDOS is derived from the Fourier transform of the velocity autocorrelation function (VACF), γ(t):

$$V(\omega) = \int \gamma(t) \exp(-2\pi i \omega t) \mathrm{d}t \tag{S11}$$

$$\gamma(t) = \frac{\left\langle \sum_i v_i(0) \cdot v_i(t) \right\rangle}{\left\langle \sum_i v_i(0) \cdot v_i(0) \right\rangle} \tag{S12}$$

where ω is the frequency; $v_i(0)$ and $v_i(t)$ are the initial velocity of an atom and its velocity at time t, respectively. The matching degree of the VDOS at the solid-liquid interface can be expressed by the overlap:

$$\text{Overlap} = \frac{\int V_S(\omega) V_L(\omega) \mathrm{d}\omega}{\int V_S(\omega) \mathrm{d}\omega \cdot \int V_L(\omega) \mathrm{d}\omega} \tag{S13}$$

where the subscripts S and L of $V(\omega)$ represent the VDOS of the solid and liquid, respectively. The denominator normalizes the overlap by the product of the individual frequency integrals of $V_S(\omega)$ and $V_L(\omega)$, so that the overlap measures the degree of spectral shape matching between the two sides and is independent of the absolute magnitude of the VDOS. With this normalization the overlap carries units of inverse frequency, and its values are therefore reported here in units of THz^{-1}; a larger value indicates better spectral matching between the two sides. Since all overlap values in

this work are computed and compared under the same normalization and units, the relative variation of the overlap with the interaction strength is well defined and unaffected by the choice of units.

To obtain a clean vibrational spectrum free from the diffusive contribution, a continuous weighting function w(t) was introduced in the time domain to decompose the normalized velocity autocorrelation function γ(t) into vibrational and diffusive components:

$$\gamma_{\mathrm{vib}}(t)=\omega(t)\cdot\gamma(t),\quad \gamma_{\mathrm{diff}}(t)=[1-\omega(t)]\cdot\gamma(t) \tag{S14}$$

The weighting function *w(t)* approaches 1 at short times and smoothly decays to 0 at long times:

$$\omega(t)=\exp(-t/\tau) \tag{S15}$$

where the decay time constant τ was determined by fitting the long-time tail of $\gamma(t)$, such that $\gamma_{\mathrm{diff}}(t)$ exhibits the same exponential decay behavior as the $\gamma(t)$ in the long-time regime. The same cosine transform was applied to $\gamma(t)$, $\gamma_{\mathrm{vib}}(t)$, and $\gamma_{\mathrm{diff}}(t)$, yielding the total VDOS ($V(\omega)$), the vibrational VDOS ($V_{\mathrm{vib}}(\omega)$), and the diffusive VDOS ($V_{\mathrm{diff}}(\omega)$), respectively. It satisfies $V(\omega)=V_{\mathrm{vib}}(\omega)+V_{\mathrm{diff}}(\omega)$.

Method S11: Phonon heat capacity ($C_V(T)$).

The phonon heat capacity can be calculated by Eq. (S18).

$$C_V(T)=3Nk_{\mathrm{B}}\int_0^{\infty}V(\omega)\left(\frac{\hbar\omega}{k_{\mathrm{B}}T}\right)^2\frac{\exp(\hbar\omega/k_{\mathrm{B}}T)}{[\exp(\hbar\omega/k_{\mathrm{B}}T)-1]^2}d\omega \tag{S16}$$

where $C_V(T)$ is the phonon heat capacity; N is the number of atoms; k_B is the Boltzmann constant; $V(\omega)$ is the vibrational density of states; $\hbar$ is the Planck constant; ω is the frequency; T is the temperature. For Ti, N is the number of Ti atoms, each contributing three vibrational degrees of freedom. For the rigid TIP4P/2005 water, the heat capacity is normalized to the six quadratic degrees of freedom per molecule (three translational and three rotational), since the intramolecular vibrations are constrained

and do not contribute. The heat capacity is reported per mole of atoms for Ti and per mole of molecules for water.

Method S12: Envelope decay time (τ_{env}) and phonon lifetime (τ_{ph}).

The envelope decay time τ_{env} was obtained from the decay of the normalized VACF envelope $\gamma(t)$, characterizing the overall coherence time of the interfacial atomic vibrations:

$$\tau_{\mathrm{env}} = -\frac{t}{\ln \gamma(t)} \tag{S17}$$

For the interfacial Ti, the characteristic decay time was additionally evaluated from the VDOS linewidth. The main spectral peak was fitted with a Lorentzian function:

$$V(\omega) = \frac{A}{1+\left(\frac{(\omega-\omega_0)}{\Delta\omega}\right)^2}, \quad \tau_{\mathrm{ph}} = \frac{1}{2\pi \cdot \Delta\omega} \tag{S18}$$

where ω_0 is the peak center frequency, $\Delta\omega$ is the half width at half maximum (HWHM), and A is the peak amplitude. The full width at half maximum of this Lorentzian form is $2\Delta\omega$. The extracted τ_{ph} corresponds to the amplitude (coherence) relaxation time associated with the decay of the velocity autocorrelation. For the interfacial Ti, ω_0 = 3.82 THz corresponds to the main Ti peak, which is spectrally well resolved, and the result is reported as the phonon lifetime τ_{ph}. For the interfacial water, the low-frequency band is overdamped: the fitted linewidth is comparable to or larger than the band-center frequency ($\Delta\omega$ on the order of several THz versus ω_0 = 1.43 THz), so the Lorentzian peak is not spectrally resolvable and a linewidth-based lifetime cannot be meaningfully defined. The characteristic time of the interfacial water is therefore taken from the time-domain envelope decay τ_{env}, which is more robust for an overdamped, liquid-like spectrum and does not rely on the existence of a resolvable peak.

Figures

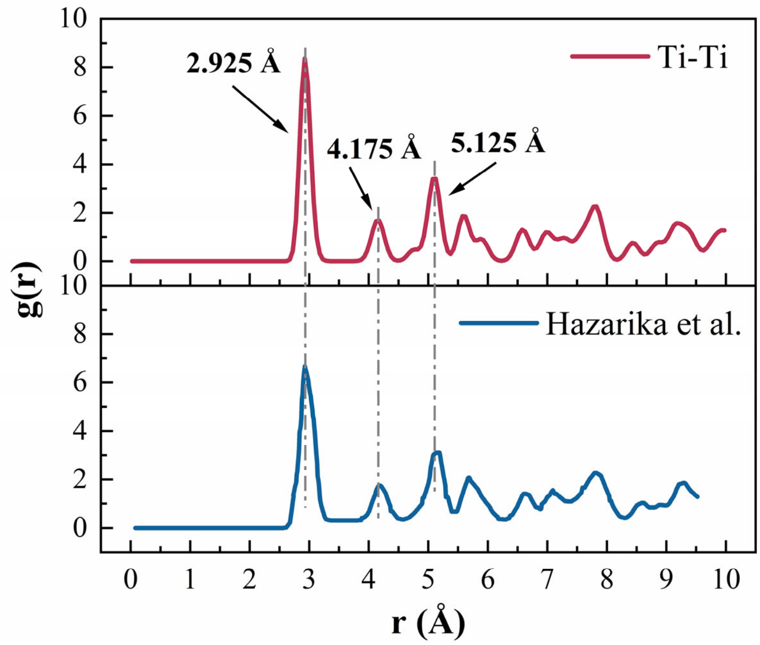

Figure S1. Comparison of the Ti-Ti RDF of the (0001) substrate model with literature data [36].

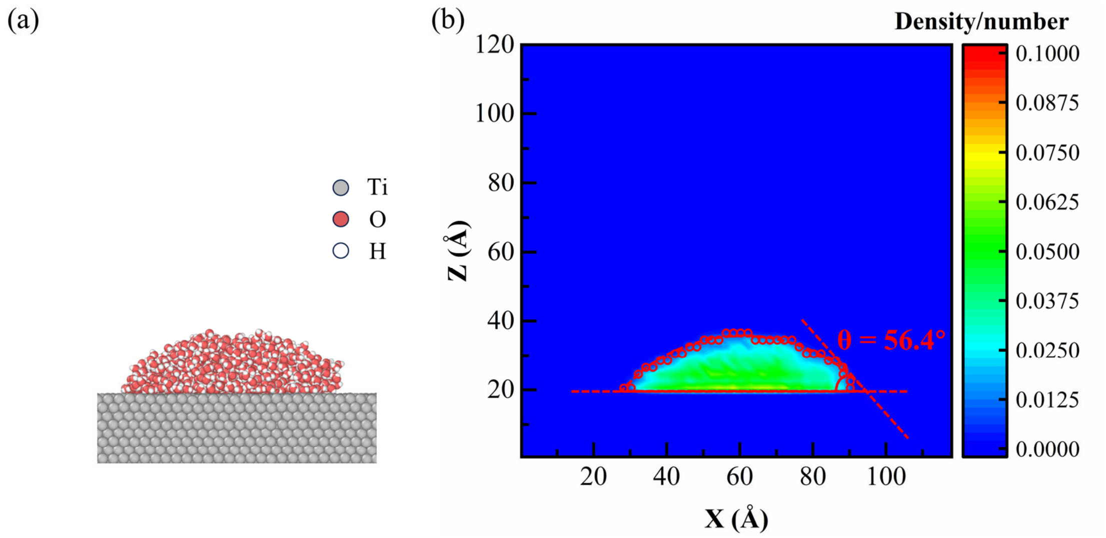

Figure S2. Determination of the water contact angle. (a) Snapshot of the equilibrated water droplet on the Ti substrate. (b) Number density distribution for contact angle fitting.

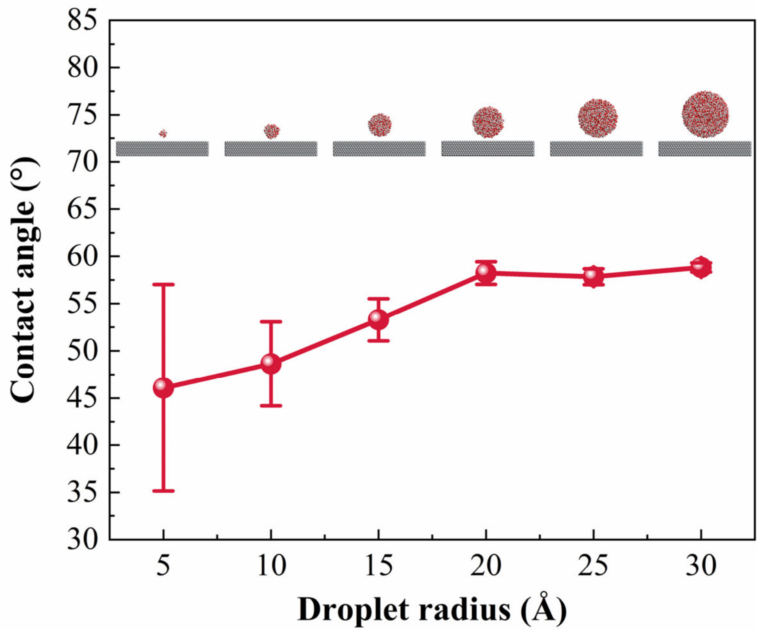

Figure S3. Contact angles of water droplets with different initial radii.

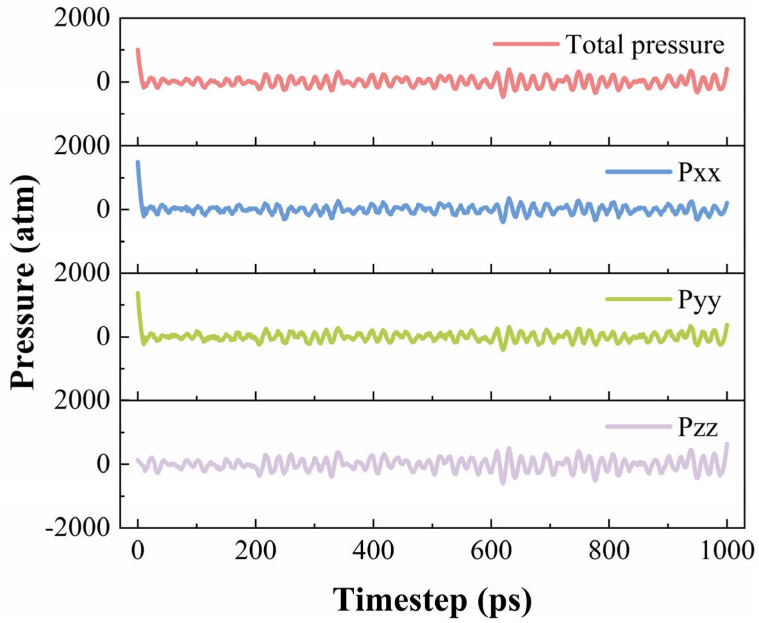

Figure S4. Pressure and stress components of Ti during equilibration.

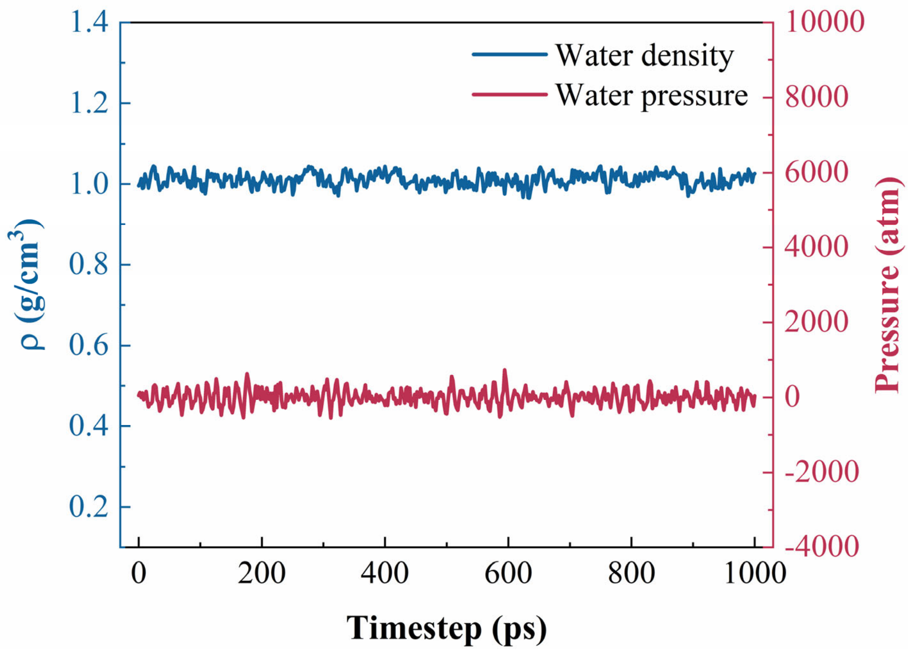

Figure S5. Density and pressure of water during equilibration.

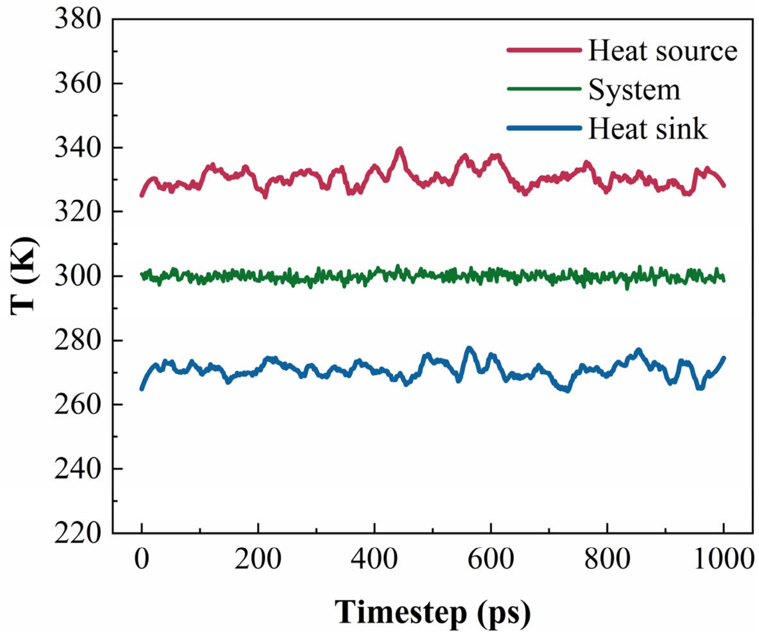

Figure S6. Temperatures of the heat source, heat sink, and system at the steady state.

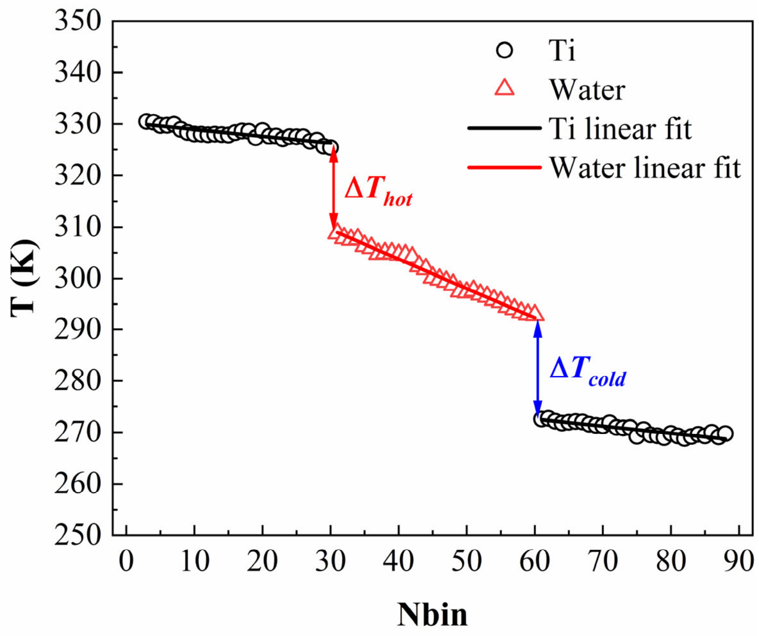

Figure S7. Temperature profile along the z direction across the solid-liquid interfaces.

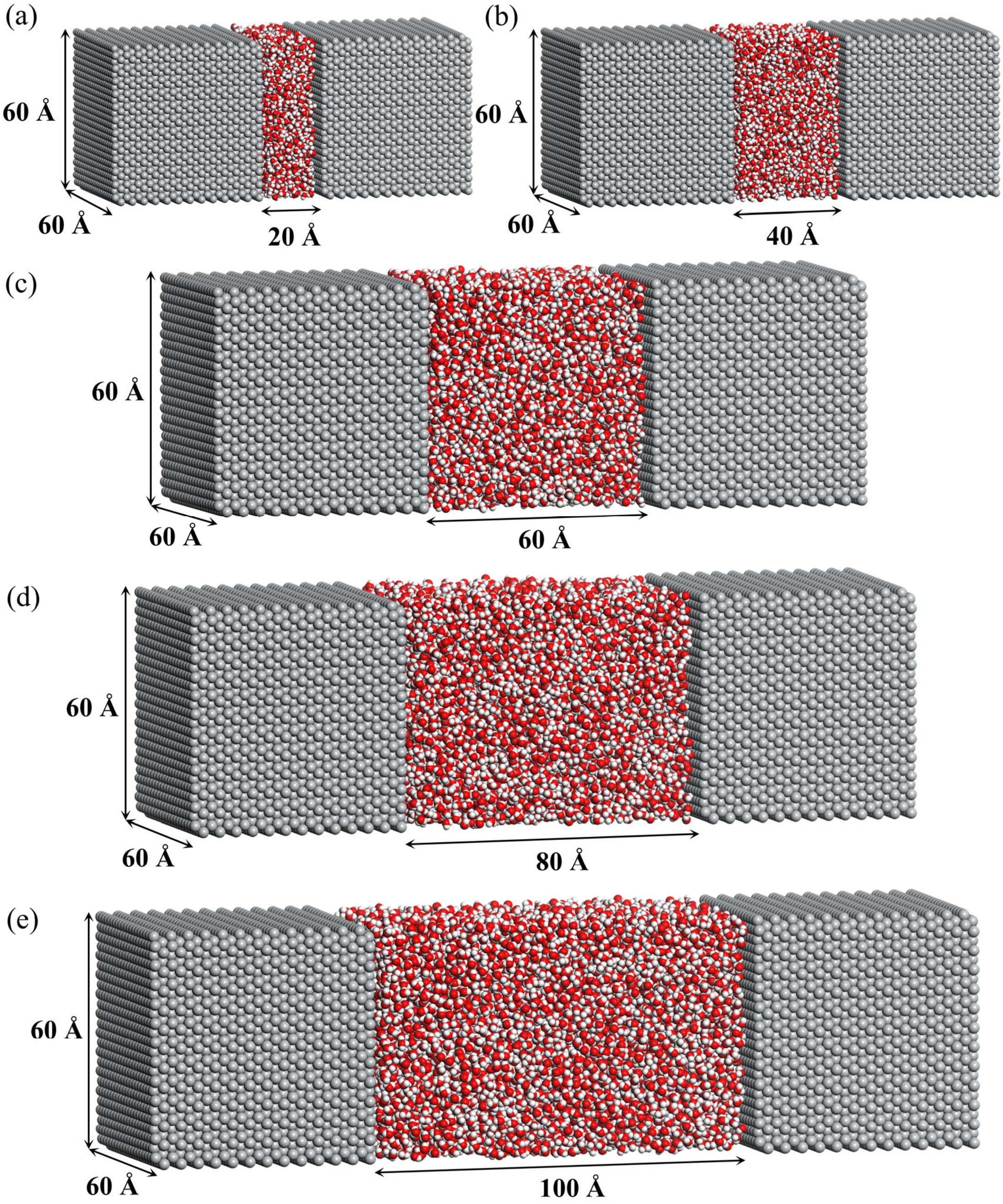

Figure S8. Heat-transfer models with different water-film thicknesses.

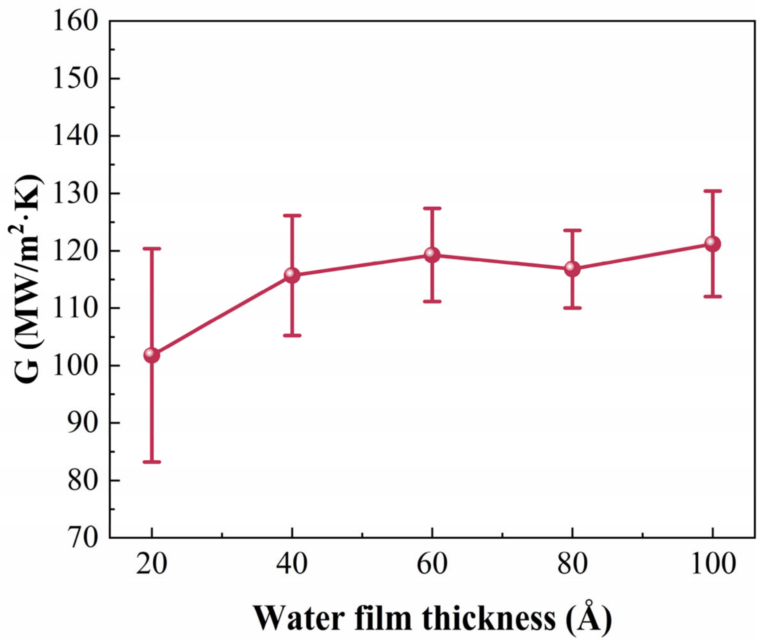

Figure S9. ITC for different water-film thicknesses at a Ti-O interaction strength of ε = 0.015 eV.

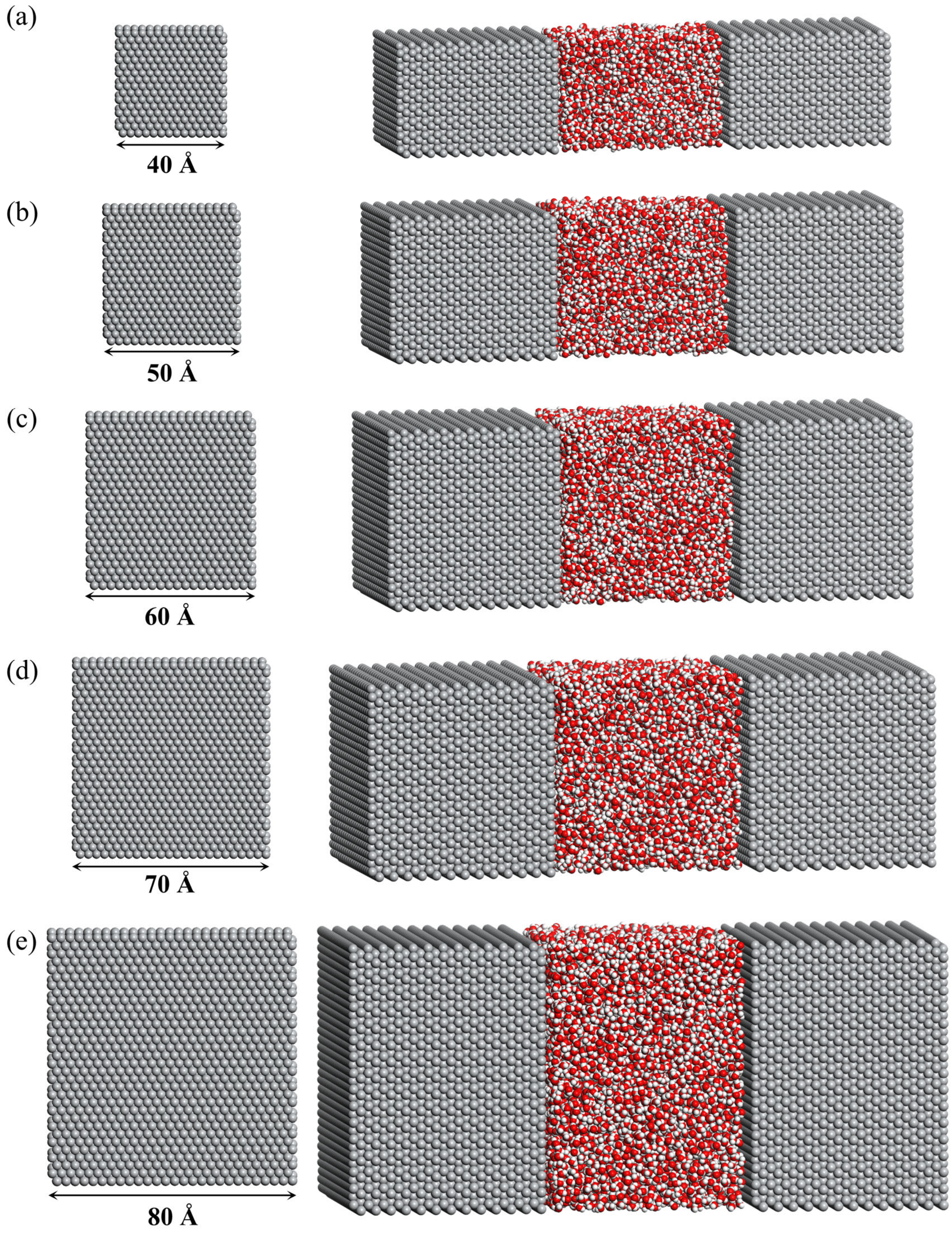

Figure S10. Heat-transfer models with different cross-sectional dimensions.

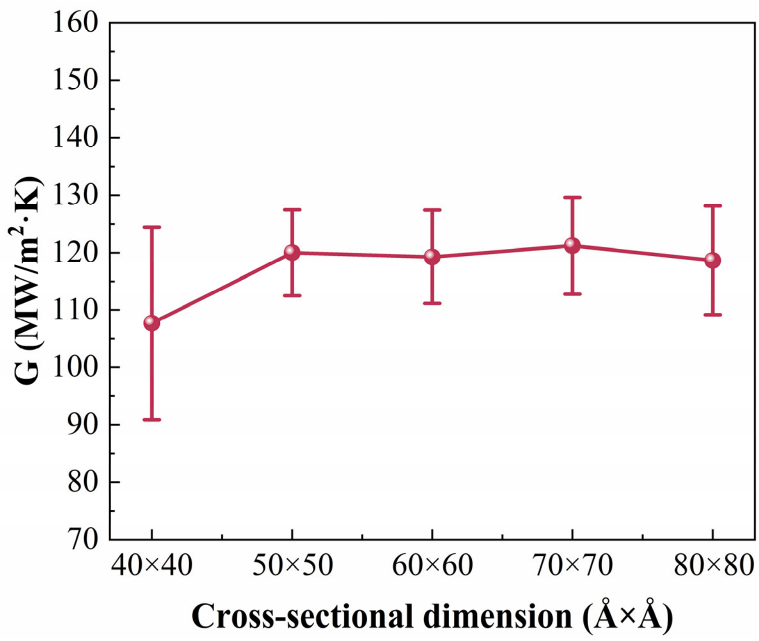

Figure S11. ITC for different cross-sectional dimensions at a Ti-O interaction strength of ε = 0.015 eV.

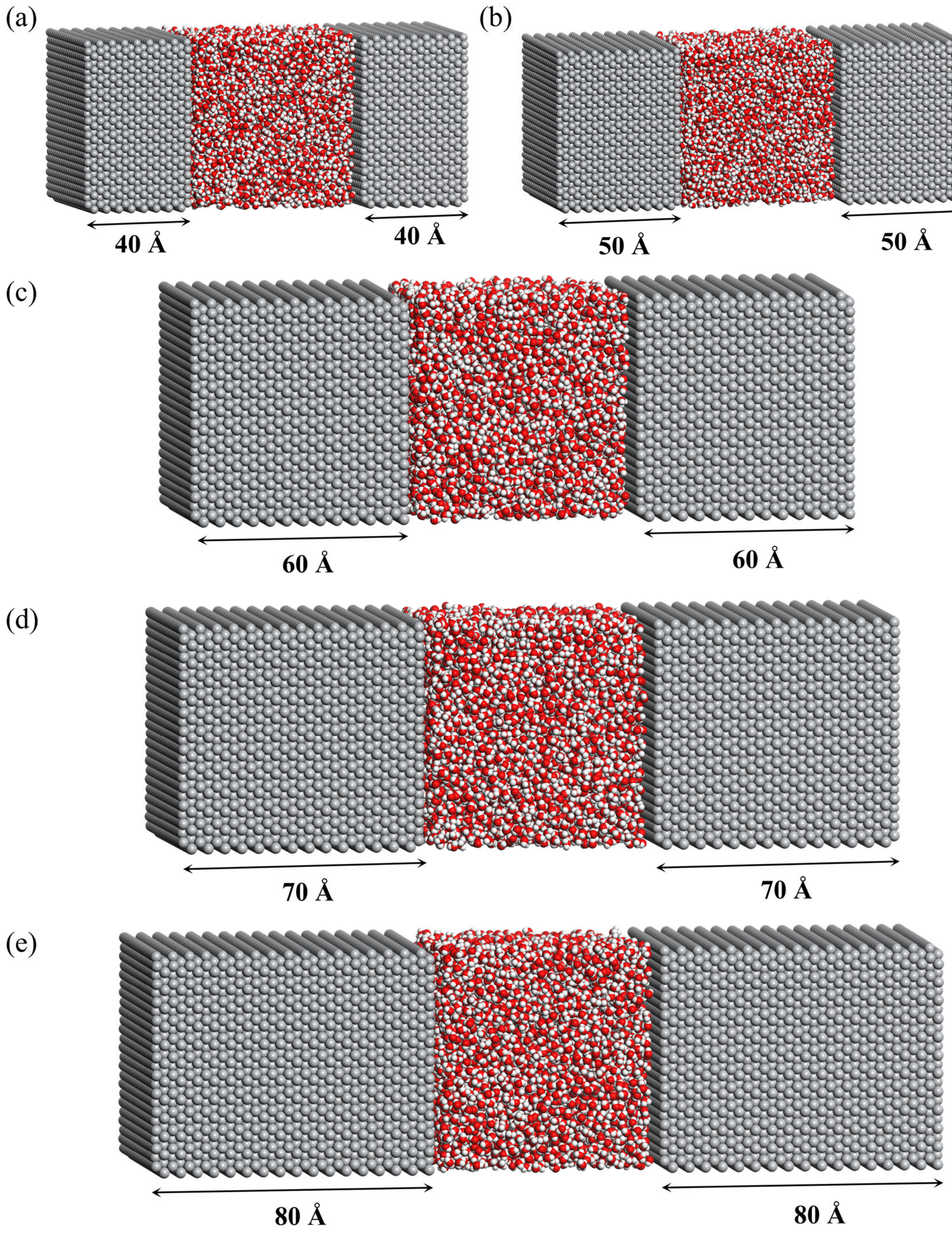

Figure S12. Heat-transfer models with different Ti-slab thicknesses.

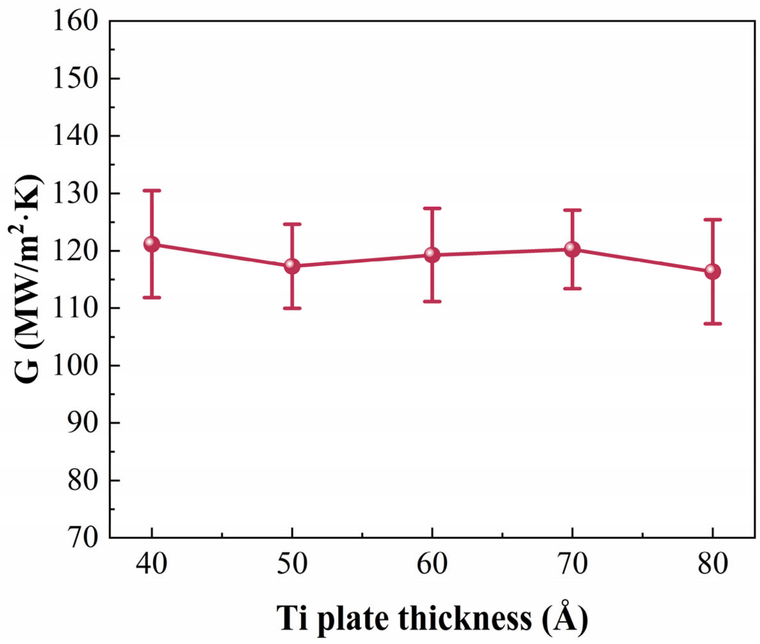

Figure S13. ITC for different Ti-slab thicknesses at a Ti-O interaction strength of ε = 0.015 eV.

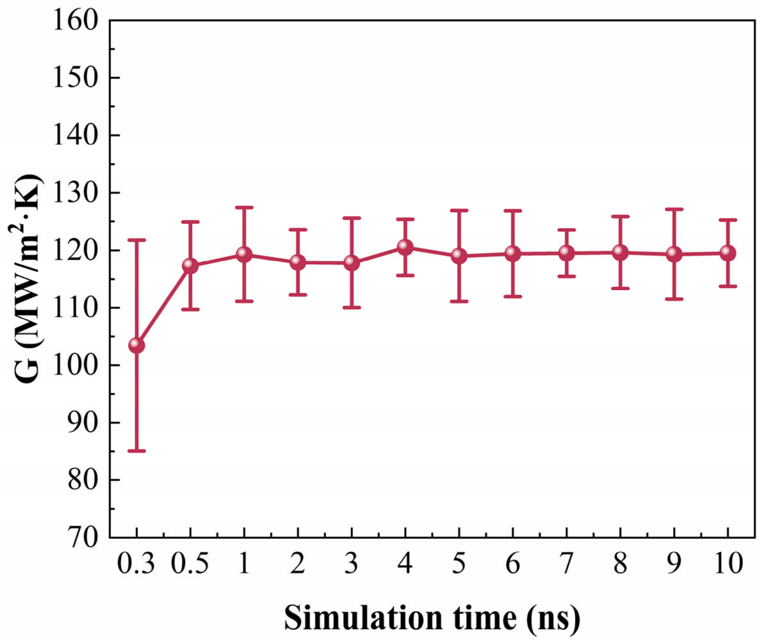

Figure S14. Convergence of the ITC with simulation time at a Ti-O interaction strength of ε = 0.015 eV.

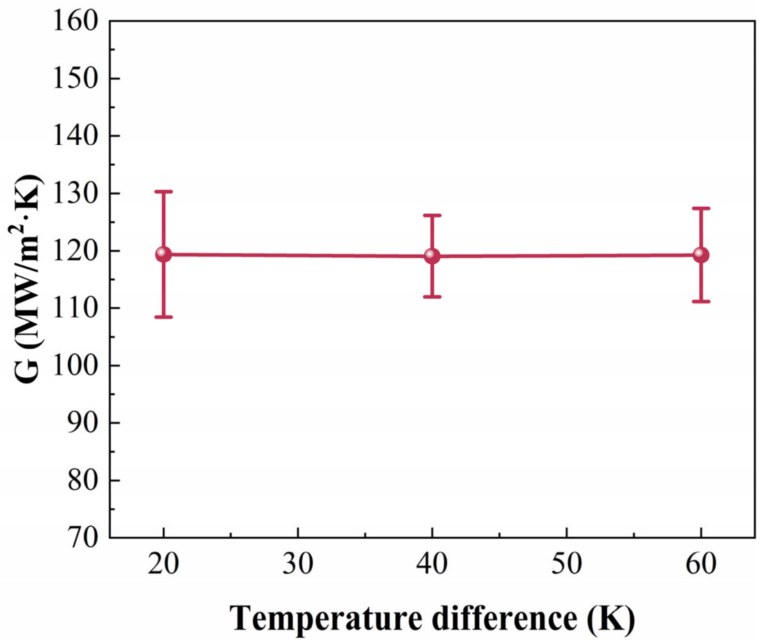

Figure S15. ITC under different imposed temperature differences. The mean temperature was fixed at 300 K for all cases, and the Ti-O interaction strength was fixed at ε = 0.015 eV.

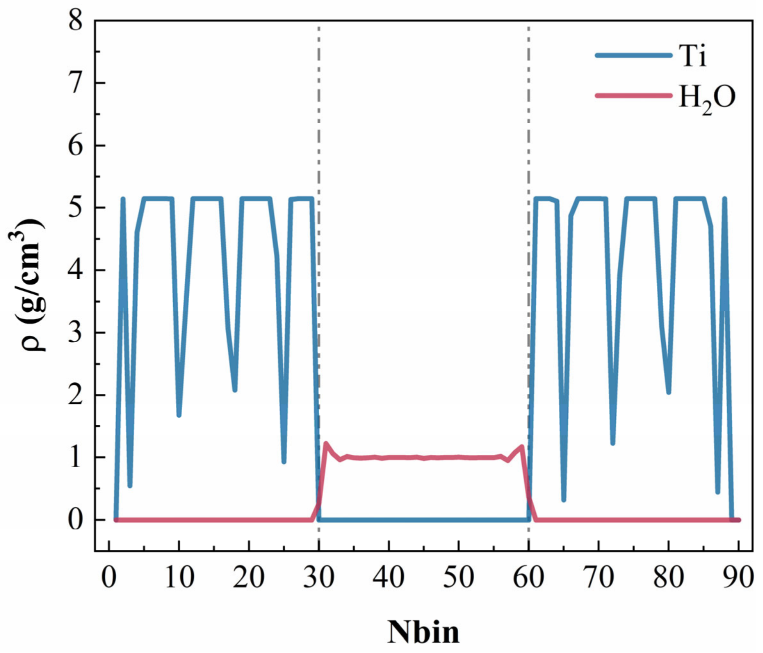

Figure S16. Density profiles along the z direction used to determine the solid-liquid interfaces.

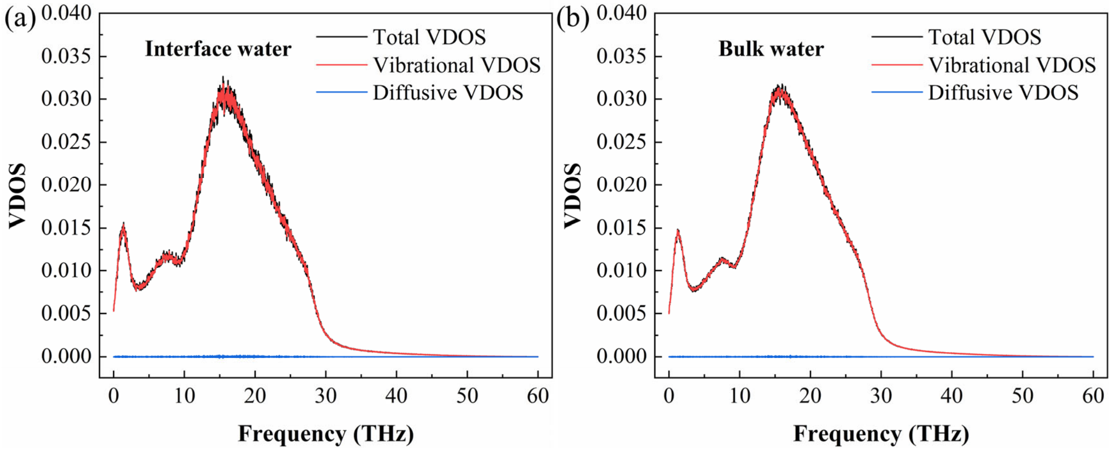

Figure S17. Separation of the diffusive contribution from the total VDOS to obtain the vibrational component. (a) Interfacial water. (b) Bulk water.

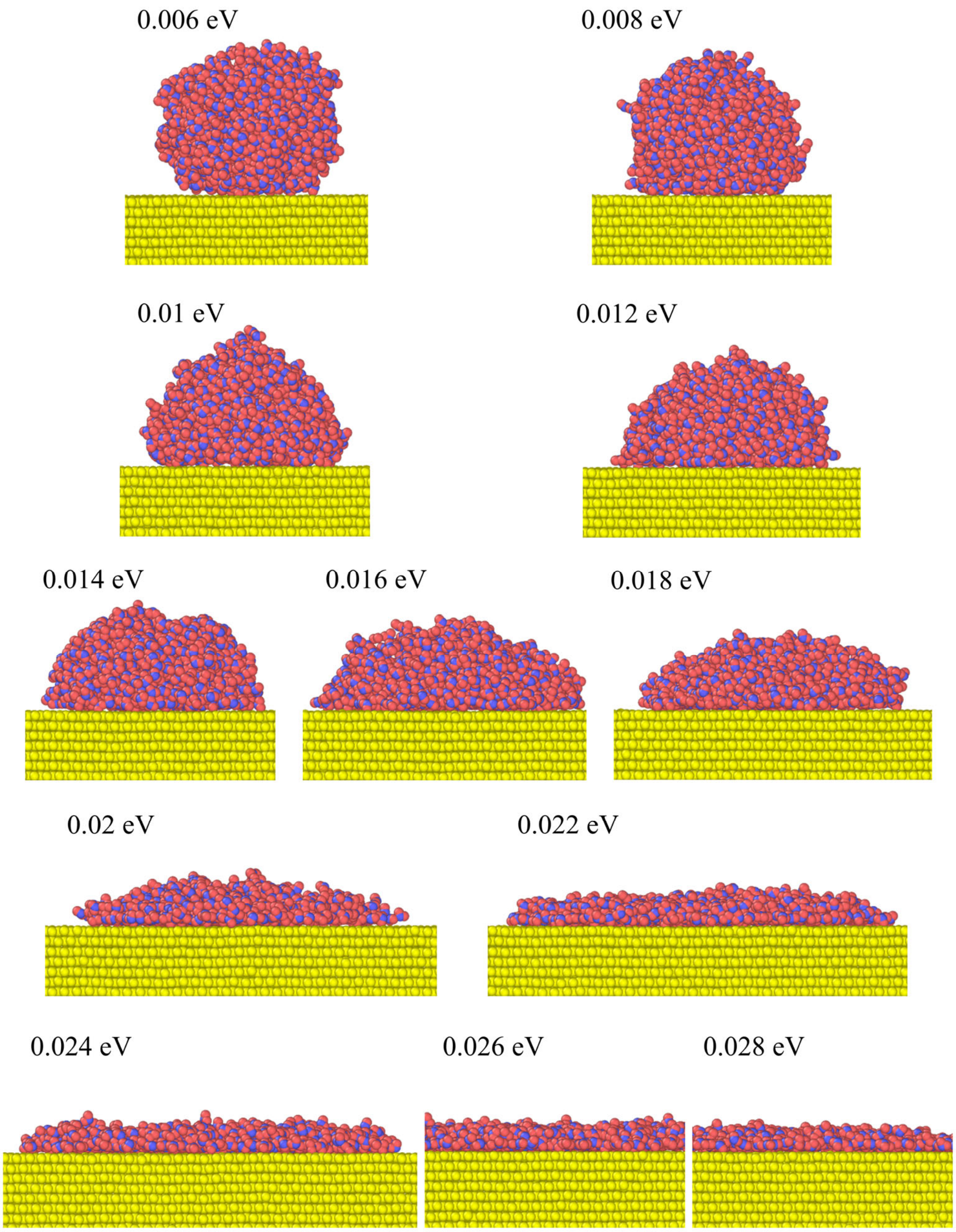

Figure S18. Side-view snapshots of the equilibrated water droplet on the Ti surface under different Ti-O interaction strengths, from the hydrophobic state to complete wetting.

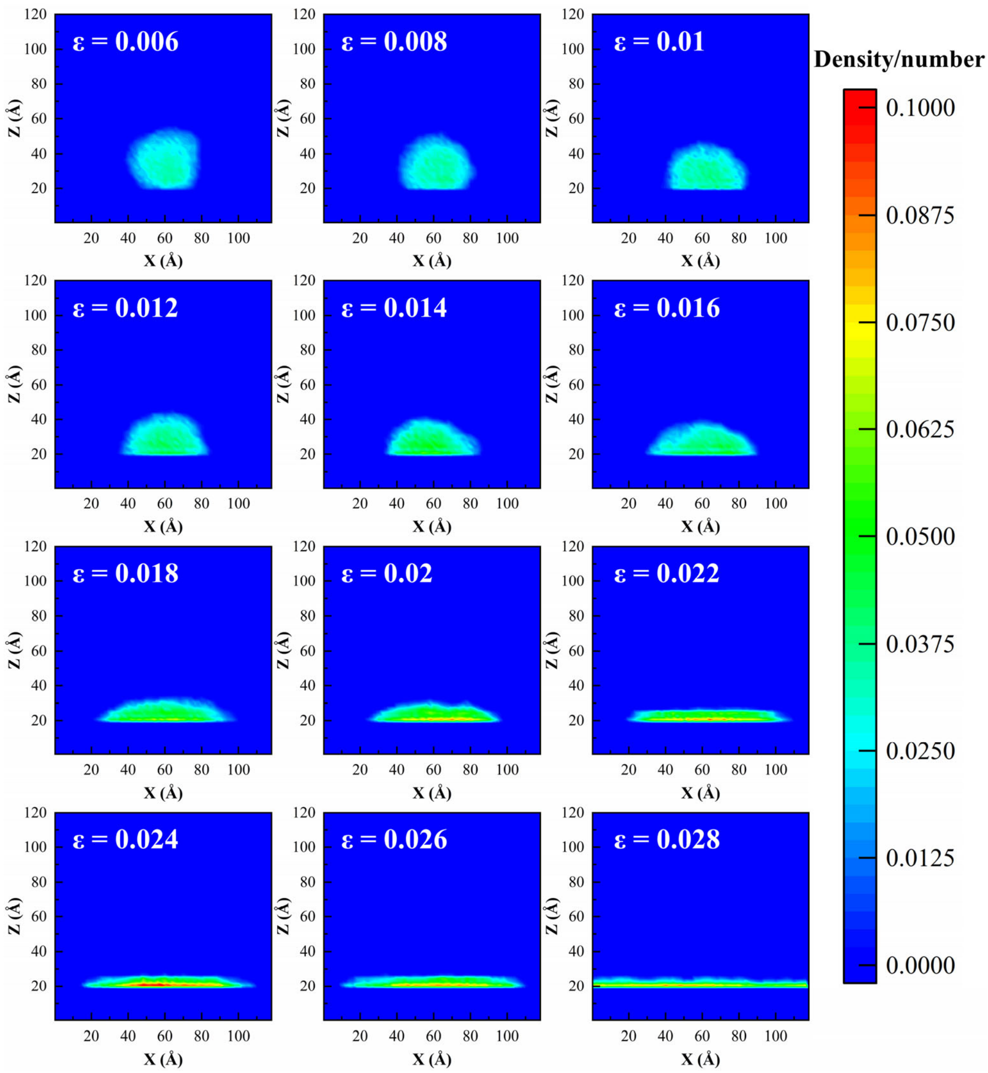

Figure S19. Number density distributions of the water droplet on the Ti surface under different Ti-O interaction strengths.

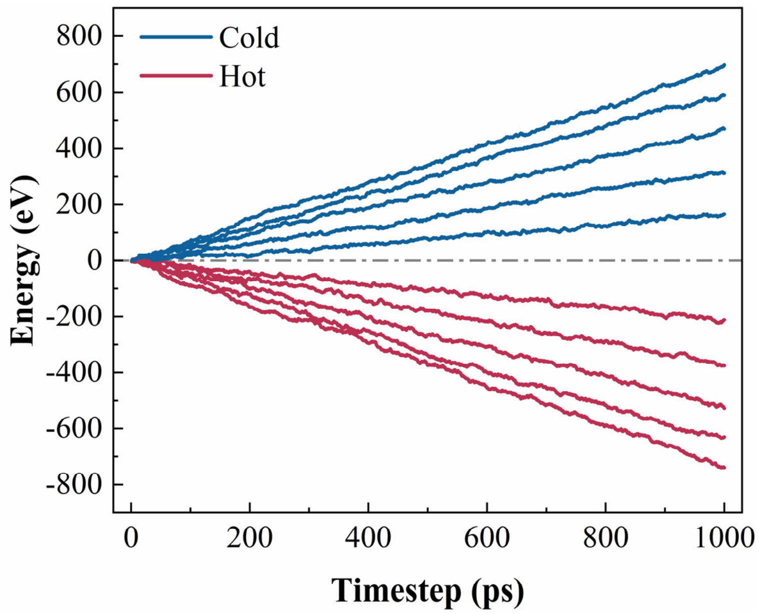

Figure S20. Energy of the heat source and heat sink versus time under different Ti-O interaction strengths.

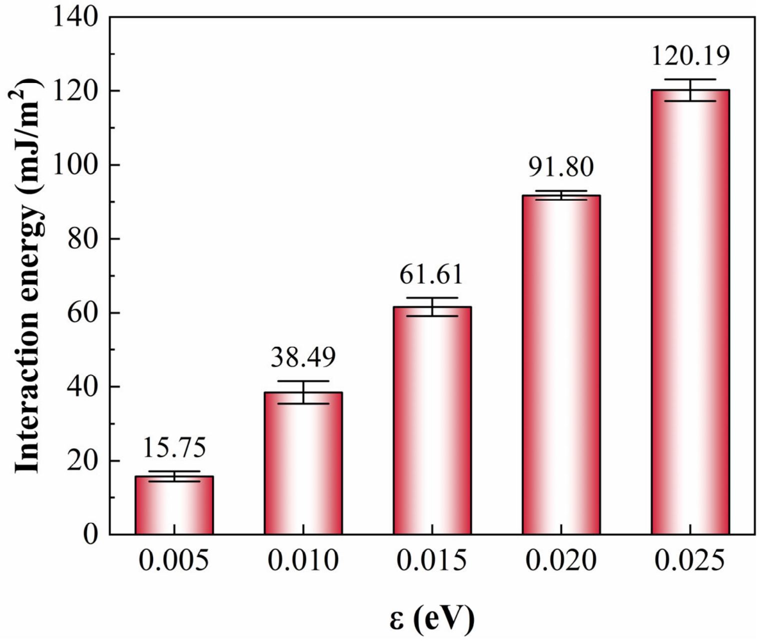

Figure S21. Interaction energy of the heat transfer model versus Ti-O interaction strength.

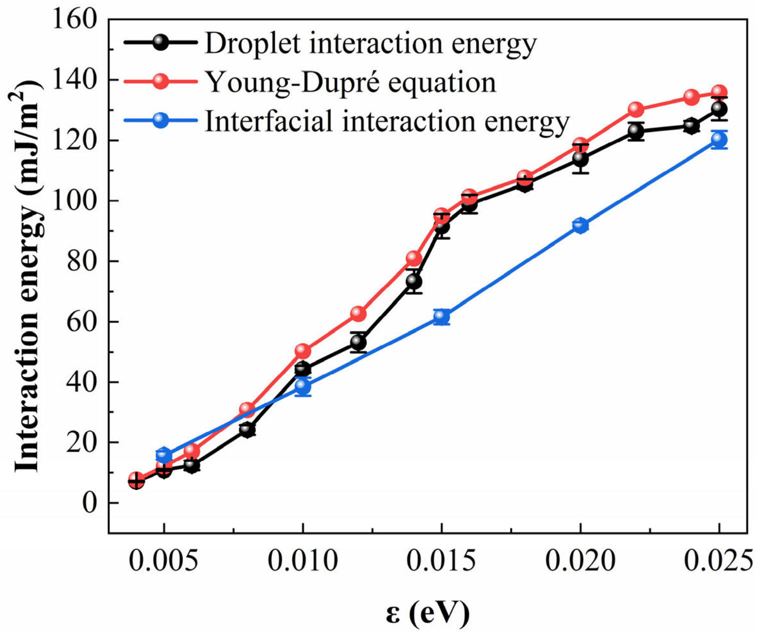

Figure S22. Comparison of the droplet interaction energy, the solid-liquid-solid interaction energy, and the Young-Dupré work of adhesion as functions of the Ti-O interaction strength.

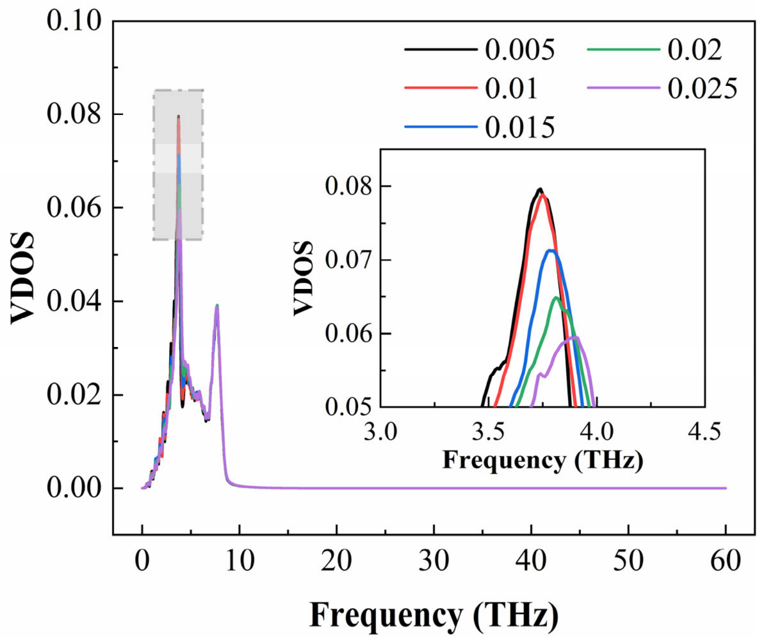

Figure S23. VDOS of the interfacial Ti under different Ti-O interaction strengths.

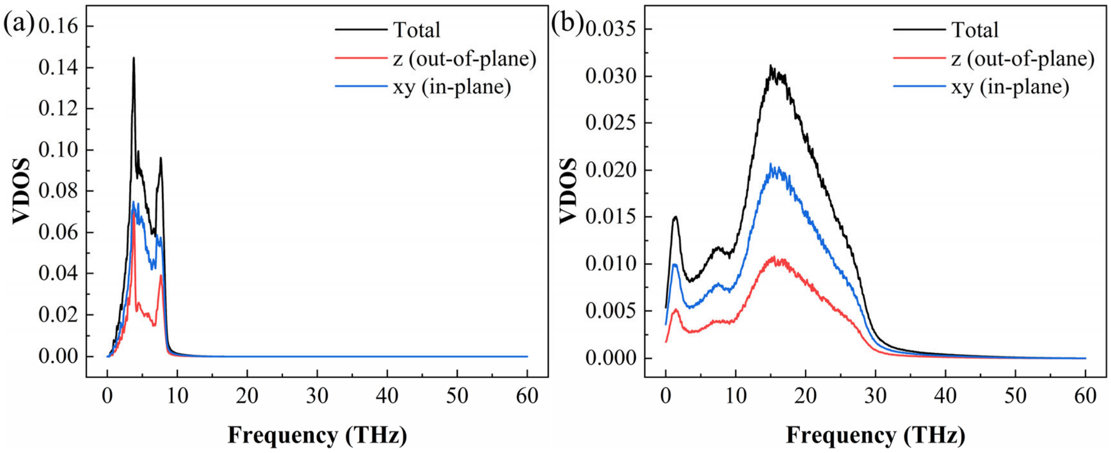

Figure S24. Directional decomposition of the VDOS into out-of-plane (z) and in-plane (xy) components at ε = 0.005 eV. (a) Interfacial Ti. (b) Interfacial water.

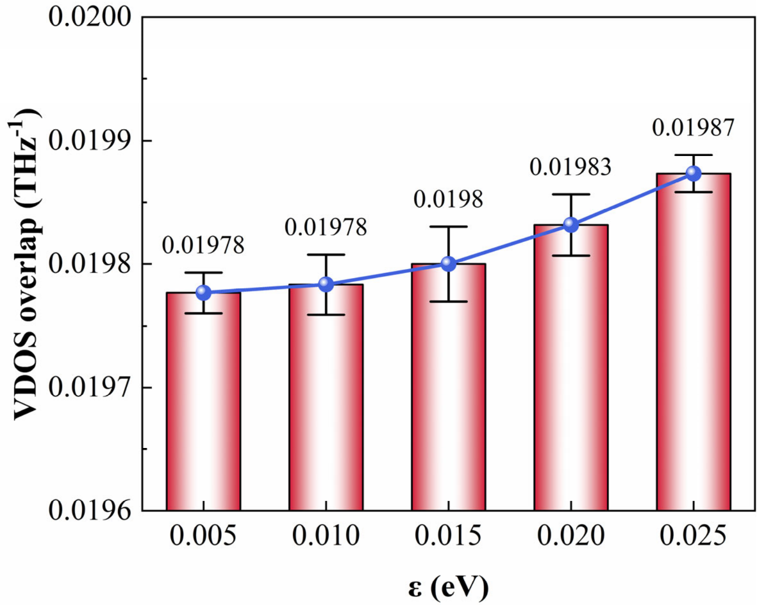

Figure S25. In-plane (xy) VDOS overlap at the solid-liquid interface under different Ti-O interaction strengths.

Tables

Table S1 LJ parameters for different atomic pairs.

Pair	ε (eV)	σ (Å)
O-O	0.00803	3.159
H-H	0	0
Ti-O	0.015	3.062
Ti-H	0	0

Table S2 The Ti-O interaction strengths used in the droplet model and the heat-transfer model.

Model	ε values (eV)
Droplet (contact angle)	0.004, 0.005, 0.006, 0.008, 0.010, 0.012, 0.014, 0.015, 0.016, 0.018, 0.020, 0.022, 0.024, 0.025, 0.026, 0.028
Heat transfer (NEMD)	0.005, 0.010, 0.015, 0.020, 0.025

Table S3 Model parameters of the heat-transfer systems with different water-film thicknesses.

Water film thickness (Å)	Water molecules	Ti atoms	Total atoms	Lz (Å)
20	2,400	25,026	32,226	140
40	4,800	25,026	39,426	160
60	7,200	25,026	46,626	180
80	9,600	25,026	53,826	200
100	12,000	25,026	61,026	220

Table S4 Model parameters of the heat-transfer systems with different cross-sectional dimensions.

Cross-sectional dimension (Å×Å)	Water molecules	Ti atoms	Total atoms	Lz (Å)
40×40	3,200	11,648	21,248	180
50×50	5,000	17,680	32,680	180
60×60	7,200	25,026	46,626	180
70×70	9,800	34,944	64,344	180
80×80	12,800	44,928	83,328	180

Table S5 Model parameters of the heat-transfer systems with different Ti-slab thicknesses.

Ti plate thickness (Å)	Water molecules	Ti atoms	Total atoms	Lz (Å)
40	7,200	16,320	37,920	140
50	7,200	20,160	41,760	160
60	7,200	24,960	46,560	180
70	7,200	28,800	50,400	200
80	7,200	32,640	54,240	220

Table S6 The droplet interaction energy, the solid-liquid-solid interaction energy, and the Young-Dupré work of adhesion $\gamma_{lv}(1 + \cos\theta)$ at the five heat-transfer interaction strengths.

ε (eV)	Droplet interaction energy (mJ/m^2)	Solid-liquid-solid interaction energy (mJ/m^2)	$\gamma_{lv}(1 + \cos\theta)$ (mJ/m^2)
0.005	10.9	15.76	12.1
0.010	44.37	38.49	50.26
0.015	91.58	61.61	95.02
0.020	113.8	91.81	118.36
0.025	130.29	120.19	135.61
